# Therapeutic application of nicotinamide: As a potential target for inhibiting fibrotic scar formation following spinal cord injury

**DOI:** 10.1111/cns.14826

**Published:** 2024-07-07

**Authors:** Ce Zhang, Qiang Shao, Ying Zhang, Wenjing Liu, Jianning Kang, Zhengxin Jin, Nana Huang, Bin Ning

**Affiliations:** ^1^ Central Hospital Affiliated to Shandong First Medical University Shandong First Medical University & Shandong Academy of Medical Sciences Jinan Shandong China; ^2^ Jinan Central Hospital Shandong University Jinan Shandong China; ^3^ School of Clinical Medicine Shandong Second Medical University Weifang China

**Keywords:** fibrotic scar, integrative analysis, metabolomics, nicotinamide, spinal cord injury, transcriptomics

## Abstract

**Aim:**

We aimed to confirm the inhibitory effect of nicotinamide on fibrotic scar formation following spinal cord injury in mice using functional metabolomics.

**Methods:**

We proposed a novel functional metabolomics strategy to establish correlations between gene expression changes and metabolic phenotypes using integrated multi‐omics analysis. Through the integration of quantitative metabolites analysis and assessments of differential gene expression, we identified nicotinamide as a functional metabolite capable of inhibiting fibrotic scar formation and confirmed the effect in vivo using a mouse model of spinal cord injury. Furthermore, to mimic fibrosis models in vitro, primary mouse embryonic fibroblasts and spinal cord fibroblasts were stimulated by TGFβ, and the influence of nicotinamide on TGFβ‐induced fibrosis‐associated genes and its underlying mechanism were examined.

**Results:**

Administration of nicotinamide led to a reduction in fibrotic lesion area and promoted functional rehabilitation following spinal cord injury. Nicotinamide effectively downregulated the expression of fibrosis genes, including Col1α1, Vimentin, Col4α1, Col1α2, Fn1, and Acta2, by repressing the TGFβ/SMADs pathway.

**Conclusion:**

Our functional metabolomics strategy identified nicotinamide as a metabolite with the potential to inhibit fibrotic scar formation following SCI by suppressing the TGFβ/SMADs signaling. This finding provides new therapeutic strategies and new ideas for clinical treatment.

## Graphical abstract

A novel functional metabolomics strategy was developed to establish relationships between changes in gene expression and metabolic phenotypes, which identified nicotinamide as a potential functional metabolite that suppressed fibrotic scar formation by suppressing the TGFβ/SMADs pathway in mice after SCI.

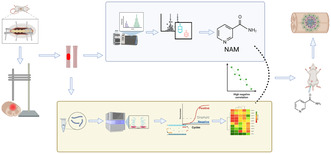

## 1 INTRODUCTION

Traumatic spinal cord injury (SCI) is a devastating disease of the central nervous system (CNS), resulting in severe locomotor dysfunction, somatosensory impairment, autonomic dysfunction, and multi‐system dysfunction,^1^, ^2^, ^3^ which seriously reduce the quality of life for patients and impose significant burden on national public health systems.^4^, ^5^ According to the staging of SCI defined by James W. Rowland et al.,^6^ the 14‐day mark falls within the subacute phase (2 days to 2 weeks), which is a crucial period for pathological changes following SCI. During this stage, M1 microglia and macrophages dominate and exacerbate inflammation through the release of inflammatory factors. Meanwhile, activated microglia and macrophages participate in the clearance of injured or dead nerve cells and myelin. Furthermore, oligodendrocyte precursor cells (OPCs) are activated and begin to differentiate into new oligodendrocytes, and a small number of OPCs can differentiate into Schwann cells. Endothelial cells are also activated, and some differentiate into oligodendrocytes in response to the injury. Second, based on existing research, at 14 days post‐SCI, fibrotic scars form, and fibroblast‐like cells fill the injury core and secrete large amounts of extracellular matrix (ECM) to form dense fibrotic scars.

The fibrotic scar is one of the most serious secondary injuries after SCI, and the temporal dynamics of fibrotic scar formation after SCI are complex and multistage, involving multiple processes such as inflammatory response, cell death, fibroblast proliferation, and extracellular matrix deposition. While the specific molecular mechanisms and timing are not yet fully understood, existing studies have provided a general description of its temporal dynamics. For instance, studies by Jerry Silver, Sofroniew, Soderblom, and Christian Göritz have confirmed that a dense fibrotic lesion core, known as the fibrotic scar, gradually forms at the injury epicenter within 14 days after SCI.^7^, ^8^, ^9^, ^10^ The primary cell types in the mature lesion core include fibroblast lineage cells (perivascular fibroblasts, meningeal fibroblasts), endothelial cells, fibrocytes, inflammatory cells, and ECM, with few or no neural lineage cells. Over time and with tissue remodeling, the elements of the lesion core persist in the form of fibrotic scars. During the development of the molecular technologies, the roles of fibrotic scar have been further elucidated in recent years. In the field of basic research, the function of fibrotic scars is two‐sided. The cells and ECM at the injury core can serve as a matrix for rapid wound repair and tissue replacement. Compared to substantial regeneration, the speed of wound closure allowed by fibrotic replacement is considered advantageous in other tissues. And fibrotic tissue has the ability to limit the spread of inflammation. However, this mechanism may come at the cost of losing tissue function. In the central nervous system, since the 19th century, lesion core tissue has been believed to have a long‐term negative functional impact on axon regeneration, inhibiting new axons from crossing the lesion site, resulting in persistent motor and sensory dysfunction.^11^, ^12^ Therefore, investigating the underlying pathophysiological mechanisms and identifying relevant biomarkers associated with fibrotic scar holds promise for enhancing the treatment of SCI and promoting functional recovery.

Recent advancements in metabolomics technology have garnered considerable attention due to its potential to establish connections between small‐molecule metabolites and disease interventions. Small‐molecule metabolites, the end products or intermediates of metabolic processes, can serve as potential biomarkers reflecting the functional status of biological systems. For instance, 5‐hydroxyproline has been identified as a key biomarker in acute ischemic stroke,^13^ while N1‐Methyl‐2‐pyridone‐5‐carboxamide, kynurenic acid, and 5‐and hydroxyindole acetic acid have demonstrated correlations with the progression of gouty arthritis.^14^ In early breast cancer, metabolites like glutamine, tyrosine, proline, histidine, alanine, and citrate have been associated with proliferating tumor cells.^15^ Besides their role as biomarkers, small‐molecule metabolites can directly interact with drugs to coordinate therapeutic strategies or bind with proteins to regulate various physiological functions, alter disease‐related signaling pathways, and affect disease progression.^16^, ^17^, ^18^, ^19^ Previous research has also underscored the potential of metabolites to regulate neuroinflammation and the post‐injury microenvironment following SCI. According to the research by Zeng et al., there is a disruption in purine metabolism after SCI, with significant changes in multiple genes and metabolites, which may participate in processes such as inflammation, apoptosis, and axonal regeneration following SCI.^20^ The findings of Rong et al. indicate that ursolic acid significantly reduces the expression of inflammatory factors such as NF‐κB, IL‐1β, and TNF‐α in the spinal cord of SCI mice by regulating intestinal microbiota and metabolic changes, significantly improving the neurological recovery and overall health status of SCI mice.^21^ Pang et al. used LC–MS/MS technology to reveal that the metabolites of arachidonic acid through the 5‐LOX pathway and COX‐2 pathway were significantly upregulated after SCI, such as LTC4, LTD4, LTE4, and PGE2, which may exacerbate inflammation and neuronal damage.^22^ Consequently, exploring novel therapeutic strategies through the perspective of functional metabolism holds promise for improving current clinical treatments and developing new drugs for SCI.

In this study, we innovatively employed a functional metabolomics strategy that combines transcriptomics and metabolomics analyses to characterize the functional metabolites responsible for regulating fibrotic scar formation post‐SCI. Following a multi‐omics analysis protocol, we initially utilized transcriptomics to identify significant enrichment of biological functions and pathways related to fibrotic scar formation, along with the upregulation of fibrosis‐related genes. Subsequently, targeted metabolomics analyses were performed to identify metabolites functionally associated with changes in fibrosis gene expression. Our investigations led to the identification of nicotinamide (NAM), a core component of nicotinate and nicotinamide metabolism, as the functional metabolite implicated in regulating fibrotic scar formation. NAM, a derivative of vitamin B3, has long been associated with the development, survival, and function of neurons in neurodegenerative diseases, playing roles in both neuronal death and neuroprotection.^23^, ^24^, ^25^ In this study, we confirmed the regulatory effect of NAM on fibrotic scar formation. Functional experiments demonstrated that NAM treatment significantly improved the functional prognosis of injured mice by reducing the expression levels of major fibrosis‐related genes (Fn1, Col1α1, Col1α2, Acta2, Vimentin, Col4α1) and reducing the extent of fibrotic scar tissue through downregulation of the TGFβ/SMADs pathway. Overall, by employing this novel functional metabolomics strategy, we have identified NAM as a significant small metabolite associated with SCI, with the potential to modulate fibrotic scar formation. The practical application of this functional metabolite may enhance the efficiency of clinical treatments and facilitate the discovery of new drugs for SCI.

## 2 MATERIALS AND METHODS

### 2.1 Animals and SCI model

All operations were performed according to the animal care standards of the Chinese National Institute of Health. Ethical approval for all animal testing protocols was granted by the Welfare and Ethics of Laboratory Animals Committee Central Hospital Affiliated with Shandong First Medical University (approval No. JNCHIACUC‐202114) on September 14, 2022.

Female C57BL/6J mice (6–8 weeks old; Weight 18–22 g) were purchased from Beijing Vital River Laboratory Animal Technology. The mice were housed in the pathogen‐free animal laboratory at Shandong First Medical University maintained at a temperature of 23 ± 1°C, and a 12 h light/dark cycle. They had unrestricted access to both water and food.

To induce SCI, mice were anesthetized with 3% pentobarbital (Sigma, 30 mg/kg) via intraperitoneal injection (i.p.), followed by a T9–T10 laminectomy to expose the spinal cord. SCI model was then induced using a spinal cord impactor (68,100, RWD, Shenzhen, China; 1 m/s, 2 mm depth, 1 s residence time). After suturing the incision site, mice were placed on a heated blanket for 2 h. The SCI mice were randomly assigned to one of two groups: SCI + NAM and SCI + Saline groups. Mice in the Sham group underwent laminectomy alone at the same spinal cord level and were similarly divided into two groups: Sham+NAM and Sham+Saline groups. From day 0 after SCI until the end of day 28, the Sham+NAM mice and the SCI + NAM mice received one i.p. of NAM once a day at a dose of 200 mg/kg. Mice in the Sham+Saline group and in the SCI + Saline group received the same dose of saline i.p. every day. The dosing schedule was uniformly arranged at a fixed period of time on each day to ensure the consistency and comparability of the experiments. In addition, we strictly followed the principle of aseptic operation throughout the experimental process to ensure the health of the mice and the reliability of the experimental results. To restore their autonomic voiding system, mice underwent manual bladder compression twice daily.

### 2.2 Behavioral assessment

All behavioral experiments were conducted under double‐blind conditions.

### 2.3 Basso‐Mouse Scale (BMS) score

The BMS score was used to evaluate the neurological function of the mice at a specific time point from 0 to 28 dpi. BMS scores ranging from 0 (complete paralysis) to 9 (normal mobility) represent the status of recovery of hind limb motor function after SCI in mice.^26^, ^27^ Prior to surgery, mice were initially placed in an open field daily for 5 days to acclimate to the walking environment, with 6 animals in each group. All assessments were performed by observers who were familiar with the grading rules and were unaware of the grouping.

### 2.4 Inclined plate test

To assess recovery in mice, we performed the inclined plate test from 0 to 28 dpi. Mice were placed on a rough rubber mat on a tilted plate. The head of the mouse was positioned toward the raised side, and the tail was directed toward the lower side of the tilted plate. The plate was gradually tilted in 5° increments starting from 0°, and the maximum angle at which mice could remain stable for at least 5 s without falling was recorded as the measurement value, with six animals in each group. The average of five experiments performed for each mouse was identified as the measurement value.^28^

### 2.5 Open field test

The open field test was performed using the Video Recording System (Saeons, China), which comprised a 45 cm × 45 cm × 35 cm empty chamber equipped with an autonomous infrared detection system for tracking small animal activity.^29^, ^30^ At 28 dpi, mice were positioned in the lower left corner of the chamber and allowed to move freely for 5 min, with six animals in each group. Infrared sensors recorded their movement trajectory, standing times, and walking distance. Before testing, mice were subjected to manual bladder compression to ensure empty bladders and eliminate the effect of bladder filling on experimental accuracy.

### 2.6 Rotarod test

To assess balance recovery in mice, the rotarod test was employed at 28 dpi.^27^ Mice underwent 30 min of daily training for a week before the experiment began. Each mouse was initially trained on a rotating rod (XR‐6C, mouse, Shanghai Xinruan) at a slow constant speed (4 rpm) for 5 min. The device was then set to an initial rate of 4 rpm with an acceleration rate of 20 rpm/min until reaching a max speed of 40 rpm. Each mouse underwent the experiment three times, with intervals of at least 20 min, and the average fall latency was recorded, with six animals in each group.

### 2.7 Footprint test

Motor function recovery at 28 dpi was further assessed using the footprint analysis, as previously described.^31^, ^32^ Red ink was applied to the front paws and blue ink to the back paws. Stride length was measured as the distance between two consecutive steps on the same side of the mouse during continuous walking, while Stride width represented the distance between the left and right feet during two consecutive steps. At least 4–6 footprints were analyzed and averaged for each mouse, with 5 animals in each group.

## 3 EXPERIMENTAL PATHOLOGY

### 3.1 Tissue preparation

At 14 dpi, mice were anesthetized with 3% pentobarbital (30 mg/kg, Sigma) and perfused transcardially with or without 4% paraformaldehyde (PFA). Tissue samples encompassing a 1.0 cm region around the injured area were carefully excised. Samples without 4% PFA were quickly frozen in cryogenic vials immersed in liquid nitrogen. These frozen tissues were used for RNA sequencing (RNA‐seq), metabolomics sequencing, RT‐qPCR, and WB. Samples with 4% PFA were dehydrated in gradient alcohols and xylene, and subsequently embedded in paraffin to prepare continuous paraffin sections, each 5 μm thick.

At 28 dpi, mice were anesthetized as described above and perfused transcardially with or without 4% PFA. Tissue samples were carefully excised as above described. Samples without 4% PFA treatment were quickly frozen in cryogenic vials immersed in liquid nitrogen, which were used for WB, RT‐qPCR. Samples with 4% PFA treatment were prepared for continuous paraffin sections as above described.

### 3.2 Hematoxylin–Eosin staining (HE)

HE staining was carried out following standard protocols. Briefly, prepared sections were deparaffinized and stained using a combination of hematoxylin and eosin (Servicebio, Hubei, China). Stained sections were dehydrated and sealed with neutral gum. Images were identified and captured using an electron microscope.

### 3.3 Masson staining

After deparaffinization, sections were sequentially immersed in solutions according to the kit instructions (Servicebio). Sections were differentiated and dehydrated, followed by transparency treatment with absolute ethanol and xylene. Neutral balsam was utilized to seal the sections, and images were observed and captured using an Olympus electronic microscope.

### 3.4 Nissl staining

Nissl staining was performed according to the manufacturer's instructions. Initially, the paraffin sections were dewaxed using an environmentally friendly dewaxing transparent liquid. Subsequently, a series of gradient alcohols were utilized to dehydrate the sections. Subsequently, the paraffin sections were treated with Nissl dye solution (Servicebio, G1036). Finally, the sections were sealed with neutral gum to ensure long‐term preservation and enhance visualization.

### 3.5 Primary mouse embryonic fibroblasts (MEFs) and spinal cord fibroblasts extraction and culture

We isolated primary MEFs using previously reported procedures.^33^ Briefly, fetal skin from pregnant mice at E12.5–14.5 was dissected and subjected to trypsin digestion at 37°C to obtain suspended cells. After removing undigested tissue using a 70 μm mesh filter, cells were collected through centrifugal sedimentation. The resuspended MEFs were seeded onto 55 mm plates (1 × 10^6^ cells/mL). MEFs were cultured in a complete medium, consisting of DMEM (High Glucose, BC‐M‐005, Nanjing BioChannel Biotechnology Co., Ltd.) and 10% Fetal Bovine Serum (BS‐1105, Inner Mongolia Opcel Biotechnology Co., Ltd.).

Newborn pups (P1/P3) were sterilized by immersion in 75% alcohol, then exposure and clipping of the entire spinal column. The spinal cord was flushed out by pushing PBS solution into the spinal canal with a 1‐mL syringe. Cut the spinal cord tissue, digest with trypsin for 15 min, and terminate digestion with a complete medium. After centrifugation, the cells were resuspended and incubated in a 10 cm dish at 37°C for 1 h, then discarding the medium. The adherent cells in the culture dish were spinal cord fibroblasts and fresh complete medium into the dish.

When cell confluence reached 80%–90%, they were passaged into 6‐well plates and subsequently treated with TGFβ (ABclonal Technology), NAM (51402ES08, Yeasen Biotechnology (Shanghai) Co., Ltd.), or SIS3 (SJ‐MX0365, Shandong Sparkjade Biotechnology Co., Ltd.).

### 3.6 Cell counting kit‐8 (CCK‐8) assay

Primary MEFs were seeded in 96‐well plates and treated with varying concentrations of NAM (0, 1, 3, 5, 7, 10, and 20 mM) for 24 h and 48 h. After treatment, cells were incubated with CCK‐8 reagent (Beyotime Biotechnology) for 2 h. Finally, the absorbance at 450 nm was measured using a microplate reader (Thermo Fisher Scientific).

### 3.7 Real‐time quantitative polymerase chain reaction (RT‐qPCR)

The RT‐qPCR primer sequences are provided in Table S3. For in vitro experiments, cells were cultured with TGF‐β or NAM treatment for 24 h, and total RNA was extracted using AG RNAex Pro RNA reagent (AG21101, Accurate Biotechnology (Hunan) Co., Ltd, Changsha, China). For in vivo experiments, tissue samples frozen in liquid nitrogen were used for RNA extraction. And the concentration of total RNA was determined using a Spectramax® Quick Drop™ spectrophotometer. Reverse transcription was performed using the reverse transcription kit (AG11728, Accurate Biotechnology (Hunan) Co., Ltd., Changsha, China). RT‐qPCR was carried out using 2 × TSINGKE® Master qPCR Mix (SYBR Green I) (TSE201, Tsingke Biological Technology) and the LightCycler®480 II Rapid Real‐time PCR System. Data were analyzed using the 2^−δδCT^ method, with all RT‐qPCR experiments performed in triplicate.

### 3.8 Western blotting (WB)

For in vitro experiments, cells were cultured with TGF‐β or NAM treatment for 48 h. For in vivo experiments, the tissue samples frozen in liquid nitrogen were used for WB. In brief, the protein was extracted as described^34^ and separated using 10% SDS‐PAGE. The proteins were then transferred to a nitrocellulose (NC) membrane and blocked using a blocking buffer solution (20 mmol/L PBS with 0.1% Tween‐20), supplemented with 5% skimmed milk at room temperature for 2 h. Universal antibody diluent (GF1600‐01) was purchased from Genefist Life Science Co., Ltd., with the dilution ratios of primary and secondary antibodies shown in Table S1. Protein expression levels were visualized using a gel imager (FluorChem M, Proteinsimple) after Sparkjade ECL super treatment (ED0015‐C, Shandong Sparkjade Biotechnology Co., Ltd.), and protein bands were quantified using Image J software.

### 3.9 Immunofluorescence (IF)

For in vivo tissue sections, samples from all groups were deparaffinized, rehydrated, washed, and subjected to sodium citrate buffer solution (pH = 6.0) at 90°C for 25 min, and permeabilized with 0.3% Triton X‐100 for 15 min. Slices were then blocked with 10% Goat Serum (C2530‐0500, VivaCell, Shanghai, China) for 1 h at room temperature (RT) and incubated overnight at 2–8°C with primary antibodies. Information on antibody dilution concentrations is provided in Table S1. The sections were further incubated with secondary antibodies for 1 h at RT and counterstained with DAPI (Beyotime Biotechnology). Anti‐fluorescence (Beyotime Biotechnology) quench sealant was added for sealing.

For in vitro experiments, MEFs were seeded on round glass coverslips in 24‐well plates, with TGF‐β and NAM treatment for 6 h and subjected to IF. Cells were fixed with 4% PFA, permeabilized in 0.3% Triton X‐100, blocked with 10% goat serum, and incubated with primary antibodies at 4°C overnight. The following day, cells were incubated with secondary antibodies for 1 h at RT and counterstained with DAPI solution.

Images were acquired using confocal laser scanning microscopy (Leica, Germany) and fluorescence microscopy (Olympus, Japan) and analyzed using ImageJ software.

### 3.10 Transcriptomics

RNA sequencing was performed by Biomarker Technologies Corporation (Beijing, China), with three animals in each group. Total RNA from spinal cord tissues was extracted using Trizol reagent, RNA concentration and purity were measured using NanoDrop 2000 (Thermo Fisher Scientific, Wilmington, DE), and the integrity of RNA was assessed using the RNA Nano 6000 Assay Kit on the Agilent Bioanalyzer 2100 system (Agilent Technologies, CA, USA). mRNA was purified from total RNA using magnetic beads, and second‐strand cDNA synthesis was performed to prepare sequencing libraries. The sequencing library was generated using the Hieff NGS Ultima Dual‐mode mRNA Library Prep Kit for Illumina (Yeasen Biotechnology (Shanghai) Co., Ltd.) and the quality of the library was evaluated on the Agilent Bioanalyzer 2100 system. The libraries were sequenced on the Illumina NovaSeq platform to obtain raw data. The data were processed using the quality control software (version) fastp 0.21.0, which removed reads with adapters, reads containing poly‐N, and low‐quality reads, while also calculating Q20, Q30, GC content, and sequence duplication levels. Subsequently, gene functional annotation, gene expression quantification, differential expression analysis, KEGG pathway enrichment analysis, and GO enrichment analysis were performed. The sequencing depth is 6G. The reference genome is Mus_musculus.GRCm38, and the Hisat2 tool software was used for alignment with the reference genome.

The GO enrichment analysis of differentially expressed genes was performed using the clusterProfiler package based on the Wallenius non‐central hypergeometric distribution.^35^ The visualization of GO analysis was completed using a bioinformatics platform at https://www.omicstudio.cn/tool.

### 3.11 Metabolomics

Metabolomics was performed by Metware Biotech Co., Ltd. following standard procedures.^18^, ^36^, ^37^ At 14 dpi, T8–T10 spinal cord tissue, including 1.0 cm around the injury site, was collected in 2.0‐mL sterile tubes, preserved in liquid nitrogen, and stored at −80°C. After being thawed and smashed on ice, the samples were mixed with 500 μL of 70% methanol/water (precooled to −20°C in advance). Samples were shaken at 280 × g for 5 min, and centrifuged for 10 min at 16,260 × g at 4°C. The supernatant was transferred to a new centrifuge tube, placed in a −20°C refrigerator for 30 min, and centrifuged at 16,260 × g for 10 min at 4°C before being transferred to a protein precipitation plate for further LC–MS analysis. Metabolite detection, identification, and quantification experiments on the collected supernatant were performed in the LC‐ESI‐MS/MS system provided by Wuhan MetWare Biotechnology Co., Ltd. Qualitative analysis of mass spectrometry data was performed using the MWDB (MetWare database). The mass spectrometry data were processed using Analyst 1.6.3 software (Sciex, Framingham, MA, USA). The mass spectrometry peak‐intensity data were collected corresponding to different concentrations of standard solutions. The integrated peak–area ratio of all detected samples was calculated by substituting the linear equation of the standard curve and then substituting the calculation formula to finally obtain the data for the substance in the actual sample.

### 3.12 T2‐weighted spinal cord magnetic resonance imaging (MRI)

At 28 dpi, both mouse groups were subjected to examination using a small animal 9.4T MRI scanner (Bruker, 9.4T Biospec, Germany). Sagittal T2‐weighted images were acquired using the Bruker ParaVision 6.0 system. Mice were anesthetized with 1.5% isoflurane (RWD, Shenzhen, China) inhalation. Scanner parameters were set as follows: T2 weighted; 320 × 320 matrix; section thickness of 0.3 mm; Echo time/Repeat time = 24/1200 ms; and turning angle of 90°.

### 3.13 Statistical analysis

For RNA sequencing, the DEGs were screened under the following conditions: |log2 Fold Change| ≥ 1 and *p*‐value <0.05. For metabolomics, the differential metabolites were screened by specific criteria (FC ≥ 1.5/ ≤0.67 and VIP ≥ 1).

Correlation analysis between metabolites and genes was performed with Spearman correlation coefficients. And the regression analysis was realized by GraphPad Prism (version 8.0.1) with a linear correlation.

The quantitative analysis of western blots was performed by ImageJ, and the operations are performed according to the imageJ standard manual (https://imagej.net/ij/docs/guide/).

For the statistics of Col1a1 immunofluorescence, we quantified the area of Col1a1+ regions.

Regarding the fluorescence intensity of GFAP and NF200, we quantified the fluorescence intensity in the selected regions (the length and width of the selected region are both 250 μm).

In our experimental design, we did adopt a double‐blind method, where observers were unaware of the group to which each sample belonged.

Differences between the two groups were evaluated using an unpaired two‐tailed Student's *t*‐test. Comparisons involving more than two groups were performed using one‐way or two‐way ANOVA. Post hoc correction tests were performed using Sidak's, Tukey's, or Dunnett's multiple comparisons test; Data normality was tested using the Shapiro–Wilk test. Value is expressed as mean ± standard deviation (SD). *p* < 0.05 was considered statistically significant. All statistical analyses were conducted using GraphPad Prism (version 8.0.1).

## 4 RESULTS

### 4.1 A functional metabolomics strategy reveals NAM as a novel metabolite attenuating fibrotic scar formation

**FIGURE 1:**
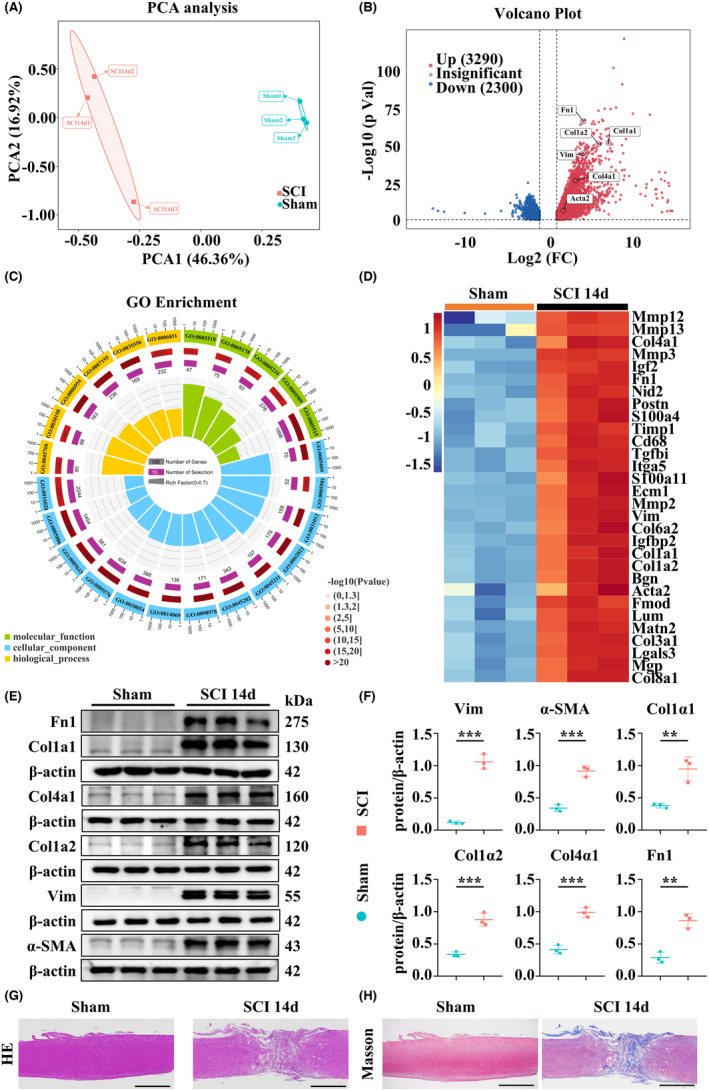
RNA sequencing and transcriptomic experiments. (A) PCA of RNA‐seq data. Each of these points represents an independent sample. (B) The volcano plot displaying the distribution of DEGs. Red points indicate significantly upregulated genes, blue points indicate significantly downregulated genes, and gray points indicate unchanged DEGs. (C) GO annotation analysis of the DEGs from the transcriptome data. There are four circles from outside to inside. The first circle: Enriched classification, outside of the circle is the coordinate scale of the number of genes, and different colors represent different classifications. The second circle: The number of genes in the classification and the *p*‐value in the background genes. The longer the bar, the more genes there are, and the smaller the *p*‐value, the redder the color is. The third circle: The total number of foreground genes. The fourth circle: RichFactor value of each classification. (D) Heatmap of fibrosis‐related DEGs among the indicated groups. (E, F) Western blotting results showing the expression of Fn1, Vim, Acta2, Col1α1, Col1α2, Col4α1 proteins. (G) HE images revealing that the structure of spinal cord tissue in the Sham group was integrated. In contrast, the structural integrity of the tissue in SCI group was disrupted, accompanied by edema, inflammatory cell infiltration, neuronal reduction, and vacuolation (scale bar = 1000 μm). (H) Masson staining showing significant deposition of fibrotic connective tissue on 14 dpi (scale bar = 1000 μm). **p < 0.01, ***p < 0.001. Significance of (F) was evaluated using an unpaired two‐tailed Student's *t*‐test.

The results of principal component analysis (PCA) revealed significant variation in the transcriptomic data between the SCI and Sham groups. This variation is primarily captured along the PCA1 axis, accounting for 46.36% of the total variability in the data (Figure 1A). Subsequently, we identified 5590 differentially expressed genes (DEGs) in the two groups, including 3290 upregulated genes and 2300 downregulated genes. To visualize the distribution of DEGs, we generated a volcano plot (Figure 1B).

To understand the biological functions of the DEGs, we performed GO annotation for DEGs. The results revealed a significant involvement of DEGs in various fibrosis processes, including collagen trimer (GO:0005581), extracellular matrix (GO:0031012), extracellular matrix organization (GO:0030198), and collagen binding (GO:0005518) (Figure 1C and Table S2). Additionally, KEGG analysis of the DEGs highlighted enrichment in pathways related to fibrosis processes (Figure S1).^38^, ^39^, ^40^, ^41^, ^42^, ^43^ Notably, we observed elevated expression levels of several fibrosis‐associated genes in SCI models compared to Sham animals (Figure 1D). To validate our RNA‐seq data, we conducted WB to measure the expression levels of several fibrosis‐related genes (Figure 1E,F). HE staining showed the fragmented structural characteristics of SCI tissue, while Masson staining revealed significant deposition of fibrotic connective tissue in the injured spinal cord (Figure 1G,H). These findings collectively indicated significant fibrotic scar formation post‐SCI.

To further explore metabolites regulating fibrotic scar formation after SCI, we employed targeted metabolomics. Orthogonal partial discrimination least squares analysis (OPLS‐DA) demonstrated a clear separation between the two groups, indicating significant changes in metabolic profiles post‐SCI (Figure S2A). The S‐Plot highlighted metabolites with notable differences between those in the upper right and lower left corners (Figure S2B). Furthermore, 200 permutation tests confirmed the accuracy of the OPLS‐DA model (Figure S2C). Meanwhile, we generated a volcano plot and identified 90 metabolites showing significant changes pre‐ and post‐injury (Figure S2D).

**FIGURE 2:**
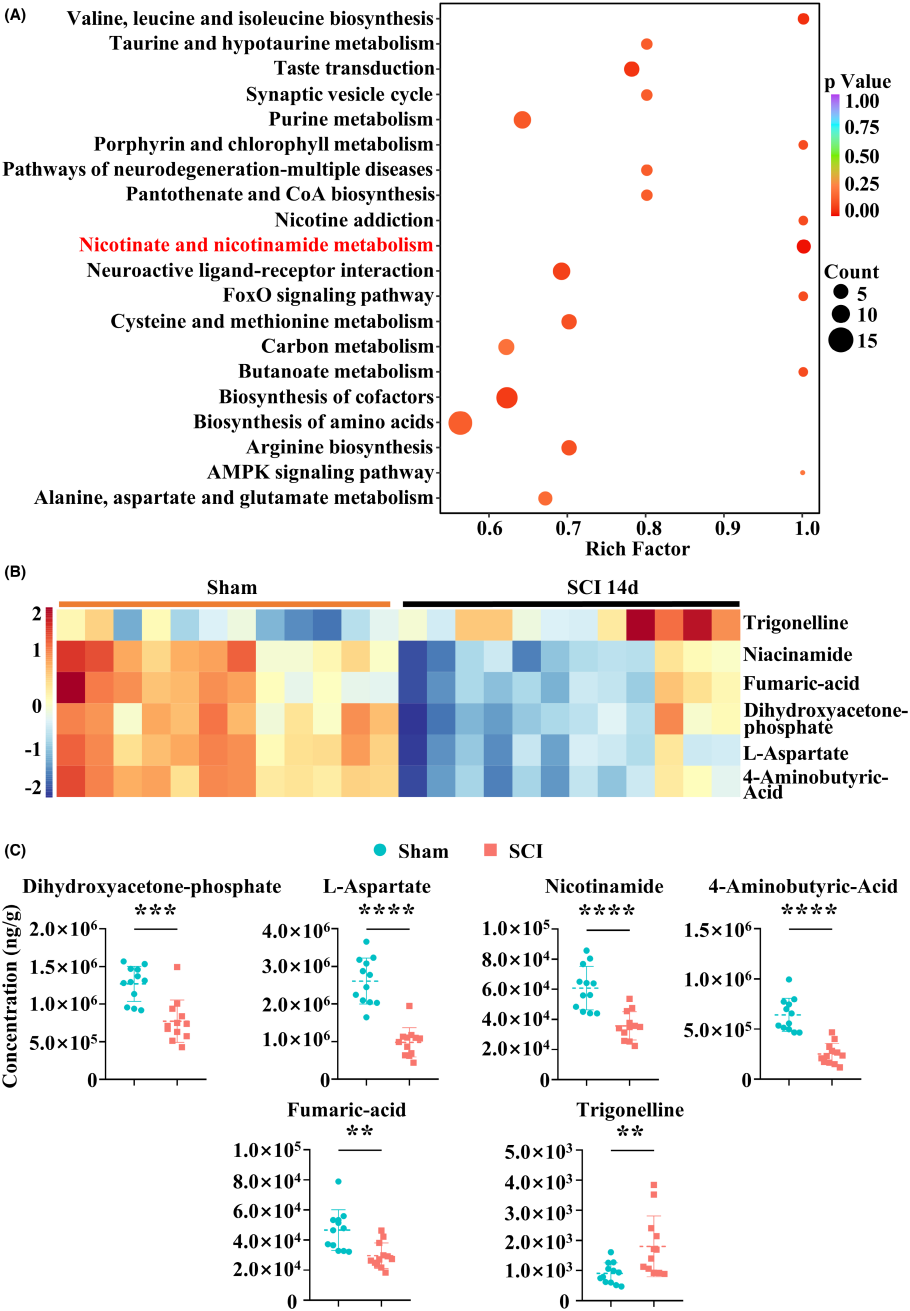
Metabolomics analysis. (A) KEGG enrichment of differential metabolites. Horizontal scales indicate the enrichment factor relative to each pathway, vertical scales indicate the pathway name, and point color indicates the *p*‐value. The size of the dots indicates the number of differentiated metabolites enriched. (B) Heatmap showing the most significant differential metabolites in nicotinate and nicotinamide metabolism pathway. (C) The dot plots showing the absolute quantitation of the selected differential metabolites. **p < 0.01, ***p < 0.001, ****p < 0.0001. Significance of (C) was evaluated using an unpaired two‐tailed Student's *t*‐test.

Additionally, KEGG enrichment analysis revealed extensive enrichment in nicotinate and nicotinamide metabolism (Figure 2A). We visualized metabolites involved in this pathway through a heatmap and quantified their levels using precisely targeted metabolomics (Figure 2B,C). This pathway is associated with various biochemical reactions, including protective effects on the nervous system,^44^, ^45^, ^46^ anti‐inflammatory effects,^47^, ^48^, ^49^ and regulation of fat metabolism.^50^, ^51^

**FIGURE 3:**
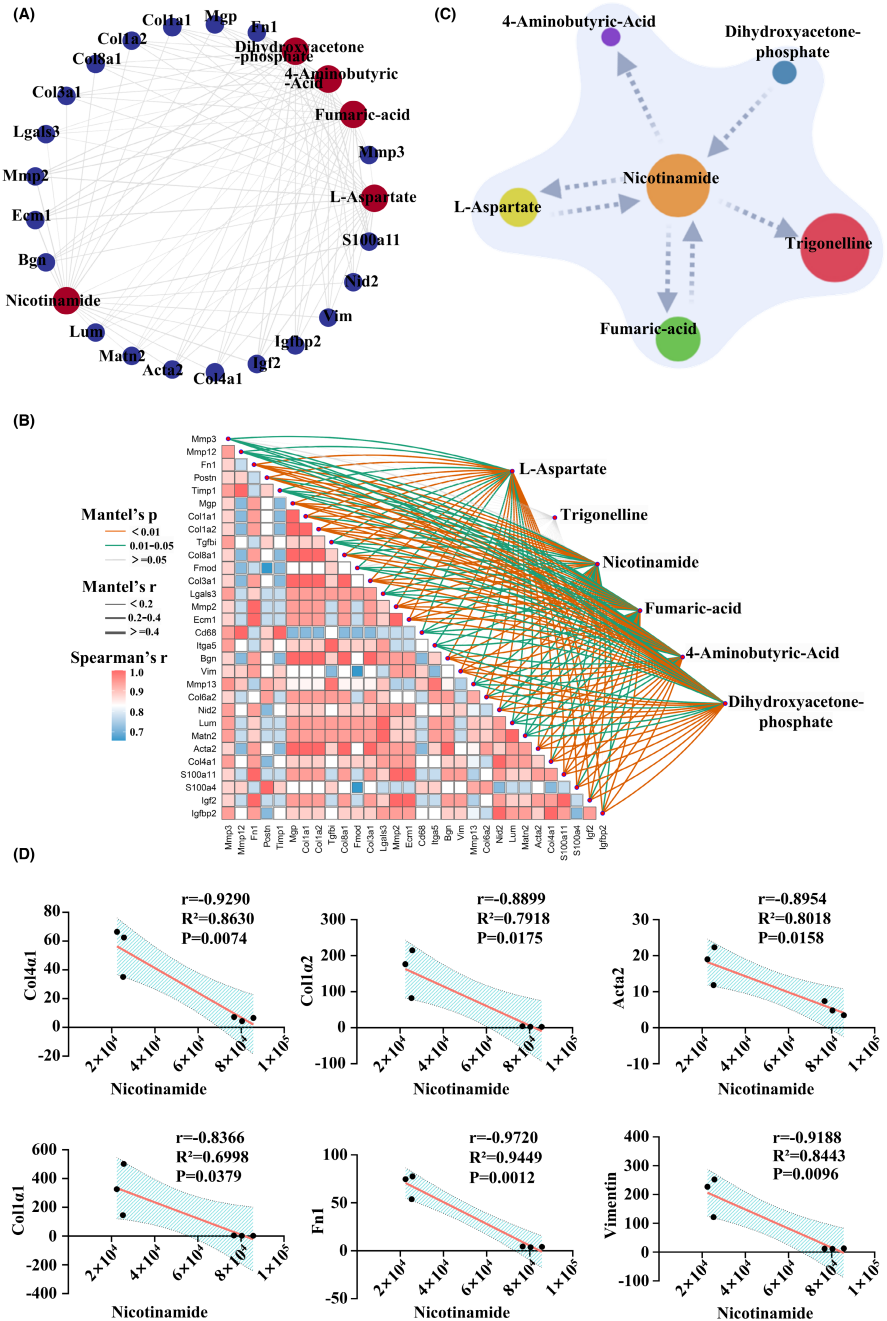
Combined multi‐omics analysis. (A) The network circle of Spearman correlation coefficient revealing that the metabolites and genes had strong interactions. (B) The correlation network heatmap showing correlations between and within omics, in which the color gradient indicates the Spearman correlation coefficient. Metabolites were linked to each gene by the Mantel's test. Edge widths represent the Mantel's r statistical measure of the corresponding distance correlation, and edge colors indicate statistical significance. (C) Pattern diagram of nicotinate and nicotinamide metabolism pathway after SCI. (D) Regression analysis revealed that NAM was strongly correlated with Col1α1, Col1α2, Col4α1, Fn1, Acta2, and Vimentin.

To determine whether the nicotinate and nicotinamide metabolism pathways can regulate fibrotic scar formation, we assessed the Spearman correlation coefficients. Results indicated that NAM exhibited significant correlation with fibrosis‐related genes (Figures 3A,B and S3). Therefore, NAM, a key molecule in nicotinate and nicotinamide metabolism, was identified as a potential regulator of fibrotic scar formation (Figure 3C). Regression analysis demonstrated a strong correlation between NAM and Col1α1, Col1α2, Col4α1, Fn1, Acta2, and Vimentin (Figure 3D).

### 4.2 NAM promotes functional recovery after SCI

**FIGURE 4:**
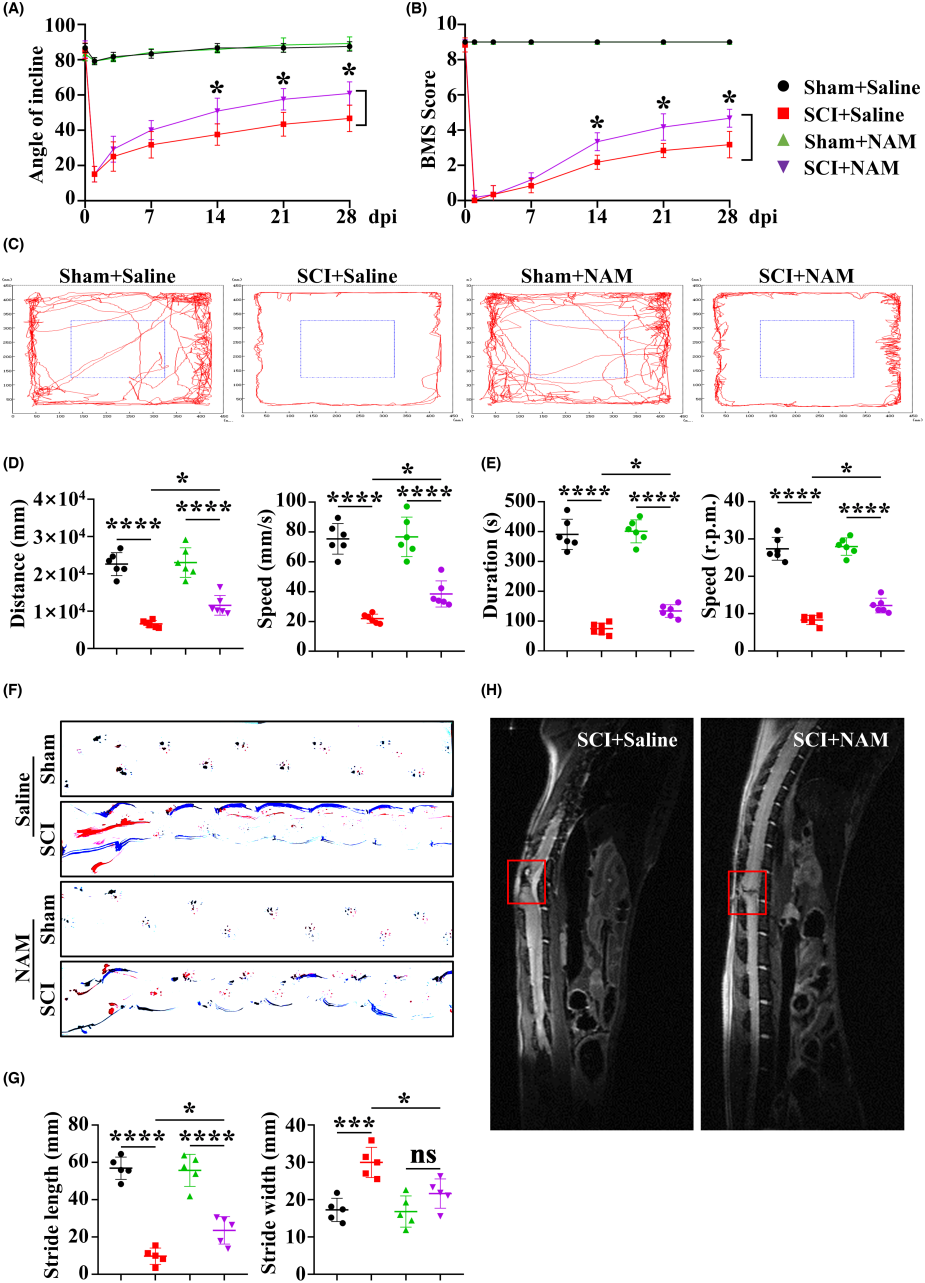
Behavioral tests reveal that NAM promotes functional recovery after SCI. (A, B) BMS and inclined plane test results on 28 dpi showed better functional recovery in SCI + NAM group compared to the SCI + Saline group (*n* = 6). (C, D) Open field test and (E) Rotarod test results on 28 dpi demonstrated better functional recovery of SCI + NAM mice (*n* = 6). (F, G) Footprint results on 28 dpi demonstrated better functional recovery of SCI + NAM mice (*n* = 5). (H) Typical images of spinal cord MRI of mice 28 dpi. The low signal intensity in the T2 phase indicated smaller hematoma area in SCI + NAM group compared to the SCI + Saline group. ns represents *p* > 0.05, *p < 0.05, ***p < 0.01, ****p < 0.0001. Significance of (A, B) was calculated using two‐way ANOVA followed by Sidak's multiple comparison test. Significance of (D, E) and (G) was calculated using two‐way ANOVA followed by Tukey's multiple comparisons test.

To assess the impact of NAM on functional outcomes post‐SCI, we established traumatic SCI models in mice and initiated intraperitoneal NAM injections from 0 to 28 dpi. We evaluated post‐SCI locomotor function recovery through BMS score, inclined plate testing, open field testing, and footprint analysis. Mice treated with NAM exhibited improved hind limb motor function at 14, 21, and 28 dpi compared to the SCI + Saline group, as indicated by higher BMS scores and inclined scores (Figure 4A,B). Open field testing for autonomous behavior and rotarod testing for hind limbs further confirmed enhanced motor functional recovery in SCI + NAM mice (Figure 4C–E). It is worth noting that although NAM showed some effects in improving motor function in mice after SCI, the results of the open field testing and rotarod testing showed no statistically significant interactions between NAM and SCI, and thus we cannot directly conclude that NAM exerts this effect by affecting SCI. We also found that the SCI mice were always only at the corners of the open field arena, indicating that SCI may induce anxiety‐like behavior in mice, and NAM treatment does not improve this anxiety‐like behavior (Figure 4C). Additionally, the footprint test revealed increased movement capabilities in NAM‐treated mice, characterized by longer stride length and reduced stride width, at 28 dpi compared to the SCI + Saline group (Figure 4F,G).

To visually observe the repair effect of NAM on spinal cord tissue after injury, the lesion area size in each group was obtained through MRI at 28 dpi. T2‐weighted MRI showed lower signal intensity in the SCI + Saline group, indicating a more extensive hematoma area and a worse hematoma extent compared to the SCI + NAM group (Figure 4H).

### 4.3 NAM alleviates fibrotic scar formation and promotes axonal regeneration

**FIGURE 5:**
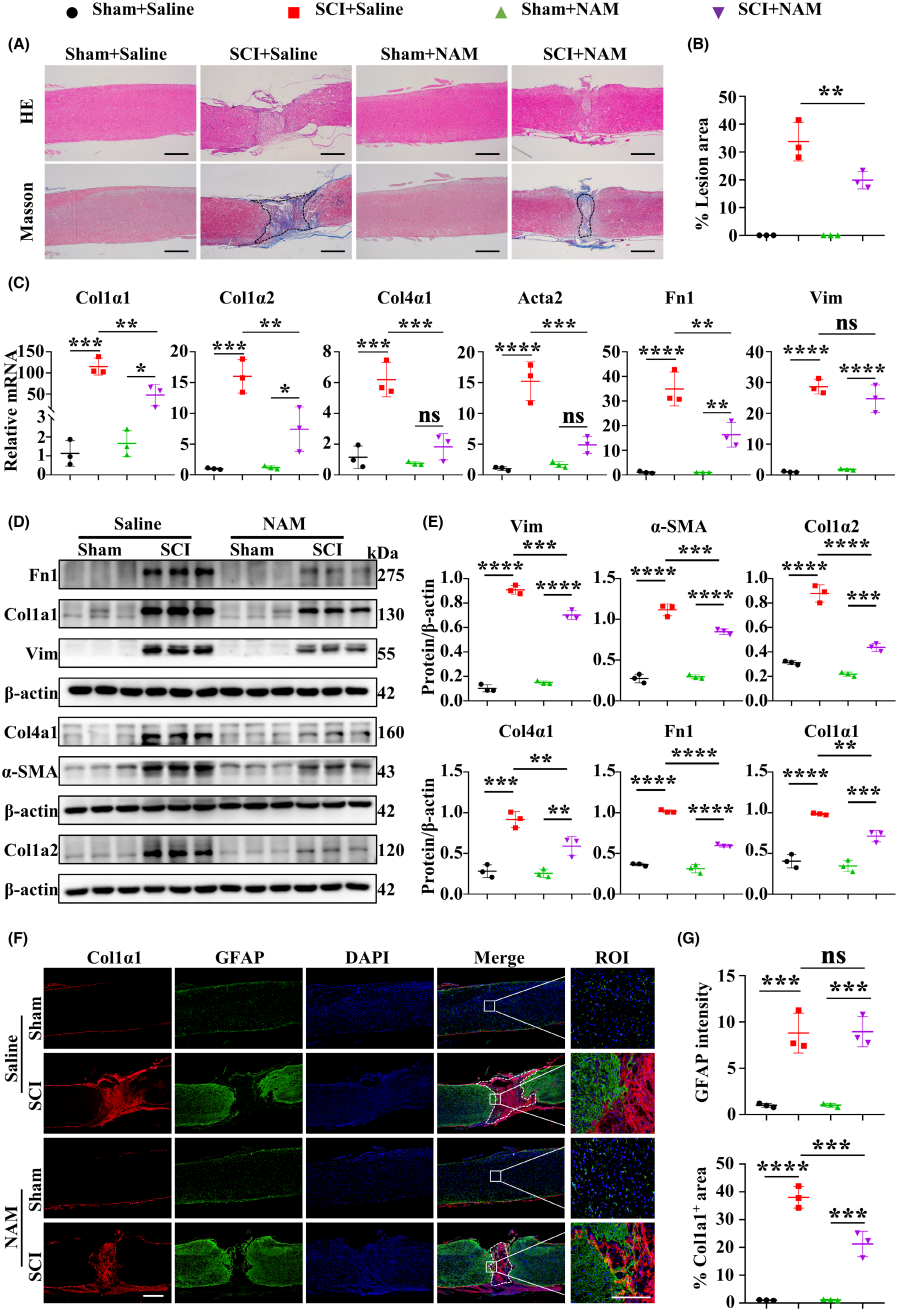
NAM alleviates scar formation after SCI in mice. (A) Longitudinal spinal cord sections collected from the indicated groups on 28 dpi were examined by HE staining and Masson staining assays (scale bar = 500 μm). (B) Quantification of Masson‐positive lesions in the spinal cords of all groups. (C) In vivo expression of Fn1, Actα2, Col1α1, Col1α2, Col4α1, and Vimentin at 28 dpi was detected using RT‐qPCR (*n* = 3). (D, E) The protein expression of Fn1, Actα2, Col1α1, Col1α2, Col4α1, and Vimentin at 28 dpi in vivo was determined by WB (*n* = 3). (F) Typical IF staining of Col1α1 and GFAP in mice spinal cord on 28 dpi (the lower left corner scale bar = 500 μm, the right left corner scale bar = 200 μm). (G) Quantification of GFAP IF intensity in zones (each the width of the spinal cord and length of 250 μm) and Col1α1^+^ area spanning the injured core at 28 dpi. NAM = 200 mg/kg/d. ns indicates *p* > 0.05, *p < 0.05, **p < 0.01, ***p < 0.001, ****p < 0.0001. Significance of (B, C, E, G) was calculated using two‐way ANOVA followed by Tukey's multiple comparisons test.

To elucidate the effects of NAM on fibrosis‐related genes, mouse spinal cord tissues were extracted at 28 dpi and assessed at both molecular and histological levels. In comparison to the Sham+Saline group, SCI + Saline displayed evident fibrotic scar formation, which was significantly reduced in the SCI + NAM group (Figure 5A,B). RT‐qPCR results demonstrated significant upregulation of Fn1, Col1α1, Col4α1, Col1α2, and Actα2 expression after SCI, with NAM treatment leading to decreased levels of these genes (Figure 5C). Notably, Vimentin expression did not significantly differ between the SCI + Saline and SCI + NAM groups. Similarly, NAM decreased the expression of Fn1, Col1α1, Col4α1, Col1α2, Actα2, and Vimentin proteins, consistent with RT‐qPCR results (Figure 5D,E). IF staining revealed no significant difference in the density of GFAP‐labeled astrocytes around the lesion site between SCI + Saline and SCI + NAM groups. However, the Col1α1^+^ area significantly decreased following NAM administration (Figure 5F,G).

**FIGURE 6:**
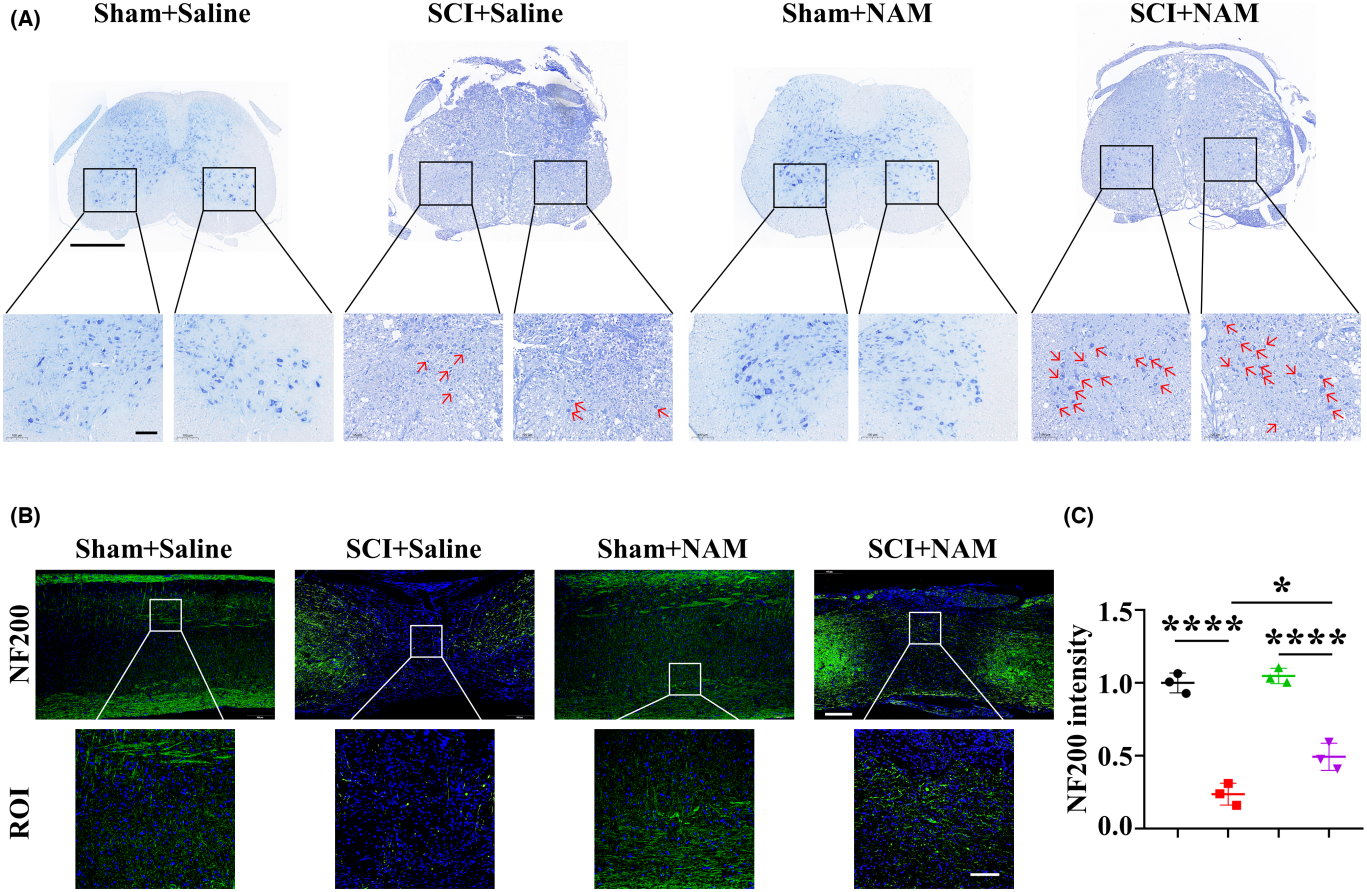
NAM inhibits neuronal cell death and promotes axonal regeneration after SCI. (A) Representative Nissl staining images in mice spinal cord on 28 dpi (the upper scale bar = 500 μm, the lower scale bar = 100 μm). (B) Representative IF images of NF200 in mice spinal cord on 28 dpi (the upper scale bar = 200 μm, the lower scale bar = 100 μm). (C) Quantification of NF200 intensity in the lesion area at 28 dpi (zone: each the width of the spinal cord and length of 250 μm). Significance of (C) was calculated using two‐way ANOVA followed by Tukey's multiple comparisons test.

The survival of neuronal cell near the lesion site was visualized using Nissl staining of the spinal cord sections that were obtained at 28 dpi. In the SCI + Saline group, the Nissl‐positive cells were significantly decreased when compared to those in the Sham+Saline group, while the SCI + NAM group showed the higher number of surviving cells versus SCI + Saline group (Figure 6A). Additionally, an increase in the IF intensity of NF200 was observed after NAM treatment (Figure 6B,C). These findings demonstrated that NAM could inhibit neuronal cell death and be involved in axonal regeneration by regulating fibrotic scar formation.

### 4.4 NAM inhibits the TGFβ/SMADs pathway after SCI in mice

**FIGURE 7:**
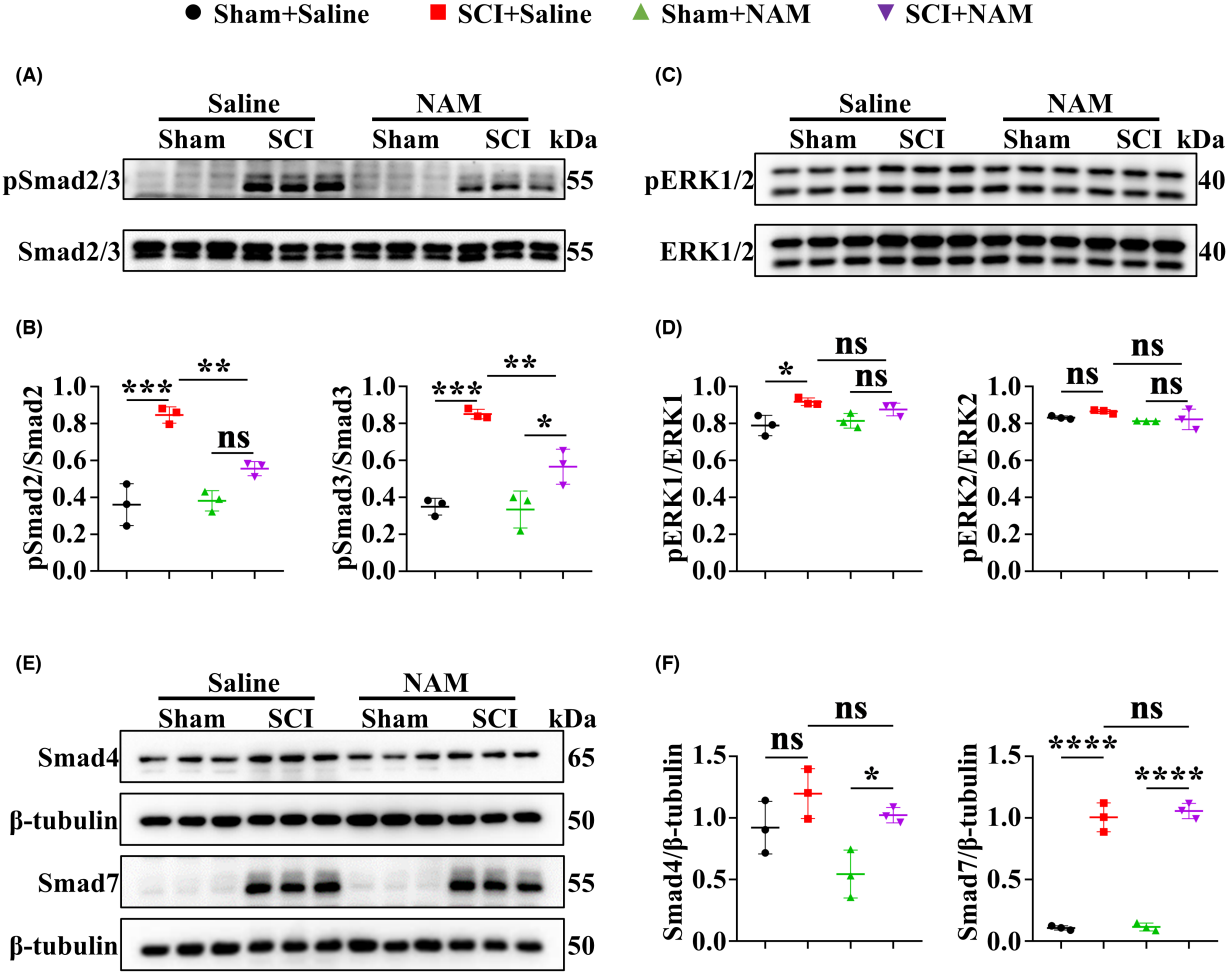
NAM alleviated scar formation mediated by the inhibition of TGF‐β/SMADs pathway in mice with SCI. (A, B) WB results showing the levels of phospho‐SMAD2/3 and SMAD2/3 proteins in vivo. (C, D) WB results showing the levels of phosphorylated ERK1/2 and ERK1/2 proteins in vivo. (E, F) WB indicating the expression of Smad4 and Smad7 proteins in vivo. ns indicates *p* > 0.05, *p < 0.05, **p < 0.01, ***p < 0.001, ****p < 0.0001. Significance of (B, D, F) was calculated using two‐way ANOVA followed by Tukey's multiple comparisons test.

Given the association of the TGFβ/SMADs pathway with fibrotic diseases, and our previous study demonstrating that inhibiting this pathway attenuates fibrotic scar formation,^52^ we investigated the mechanism by which NAM suppresses fibrotic scar formation and confirmed NAM regulated the activation of this pathway in vivo. WB results indicated an upregulation of SMAD2/3 phosphorylation following SCI, which was downregulated by NAM (Figure 7A,B). This suggests that NAM inhibits TGFβ/SMADs pathway activation by suppressing SMAD2/3 phosphorylation. The effects of NAM on other pathways involved in fibrosis were also assessed, and we found that NAM had no significant influence on phosphorylated ERK1/2 levels post‐SCI (Figure 7C,D). SMAD4 and SMAD7 have a regulating activity to SMAD2/3 and play a significant regulatory role in pathophysiological processes such as skin healing and pulmonary fibrosis; however, NAM showed no obvious effect on the protein expression of SMAD4 and SMAD7 at 28 dpi (Figure 7E,F). These findings collectively suggest that NAM suppresses TGFβ/SMADs signaling by inhibiting the phosphorylation of SMAD2/3, thereby decreasing fibrotic scar formation after SCI.

### 4.5 NAM attenuates TGFβ‐induced fibrosis in MEFs and spinal cord fibroblasts

**FIGURE 8:**
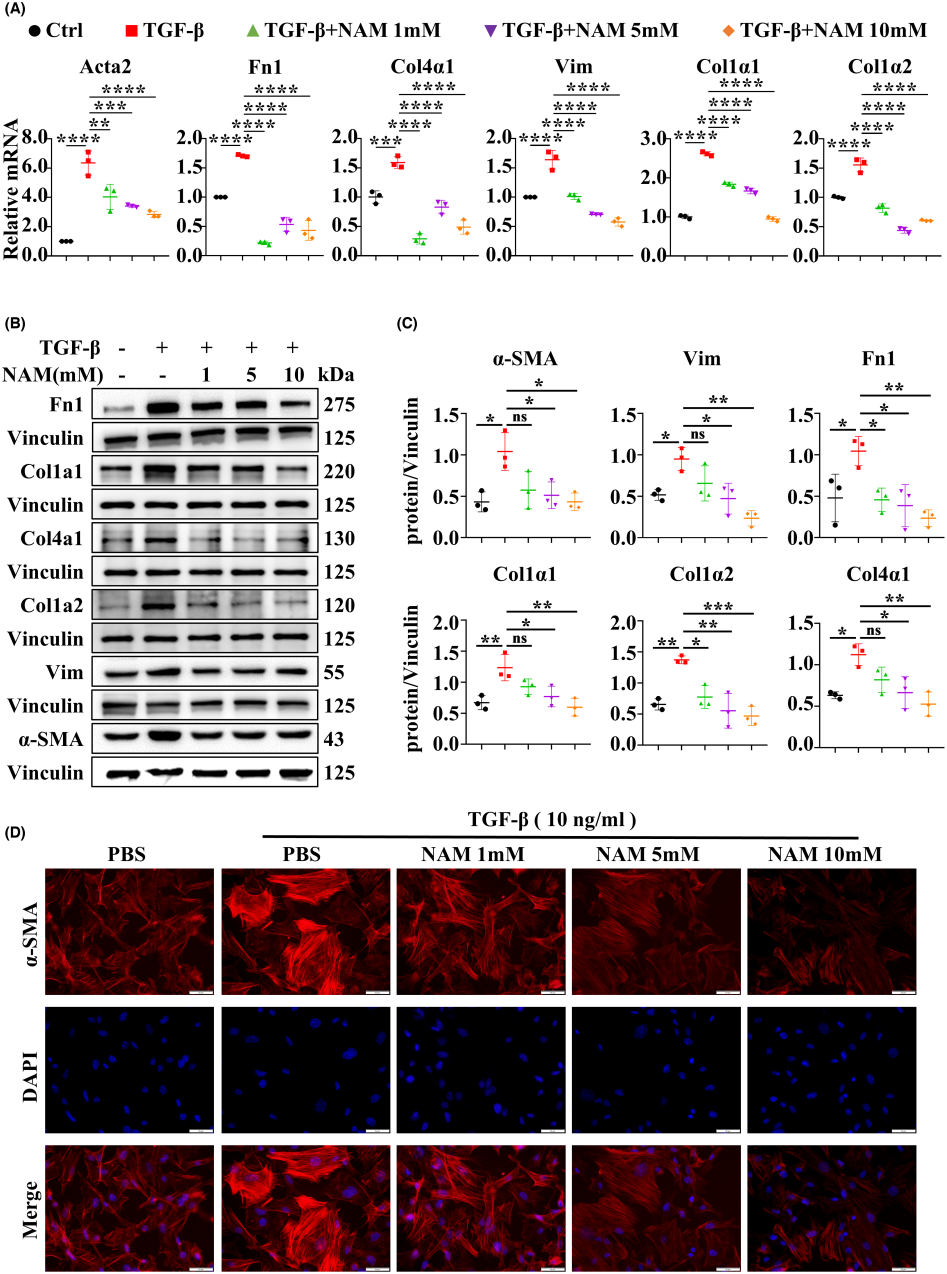
NAM inhibited fibrosis‐related gene expression induced by TGFβ in vitro. (A) The expression of Col1α1, Col4α1, Fn1, Actα2, Col1α2, and Vimentin in vitro following treatment with different concentrations of NAM for 24 h was detected by RT‐qPCR. (B, C) The results of WB showing the expression of Col1α1, Col4α1, Fn1, Actα2, Col1α2, and Vimentin proteins in primary MEFs exposed to different concentrations of NAM for 48 h. (D) Representative IF images of Col1α1 in primary MEFs with or without TGFβ stimulation and incubation with PBS or NAM for 48 h (scale bar = 50 μm). TGFβ = 10 ng/mL. ns indicates *p* > 0.05, *p < 0.05, **p < 0.01, ***p < 0.001, ****p < 0.0001. Significance of (A, C) was calculated using one‐way ANOVA followed by Tukey's multiple comparison test.

We examined the effects of seven NAM concentration gradients ranging from 0 to 20 mM on MEFs. As shown in Figure S4, CCK‐8 assays indicated that 10 mM NAM had minimal toxic effects on MEFs. To further examine the suppressive effect of NAM on fibrosis in vitro, we initially assessed the expression of fibrosis‐related genes such as Col1α1, Col1α2, Col4α1, Fn1, Acta2, and Vimentin by RT‐qPCR. The results revealed that varying concentrations of NAM could inhibit the TGFβ‐induced upregulation of fibrosis‐related genes to varying degrees (Figure 8A), and we found similar results in spinal cord fibroblasts (Figure S5). Subsequently, WB results demonstrated that the expression of Col1α1, Col1α2, Col4α1, α‐SMA, Fn1, and Vimentin increased following TGFβ treatment but decreased with the addition of 1–10 mM NAM (Figure 8B,C). IF analysis displayed that NAM significantly reduced α‐SMA intensity in a concentration‐dependent manner (Figure 8D). Based on these findings, we concluded that NAM effectively inhibits the activation of MEFs and spinal cord fibroblasts.

### 4.6 NAM inhibits TGFβ/SMADs pathway in vitro

**FIGURE 9:**
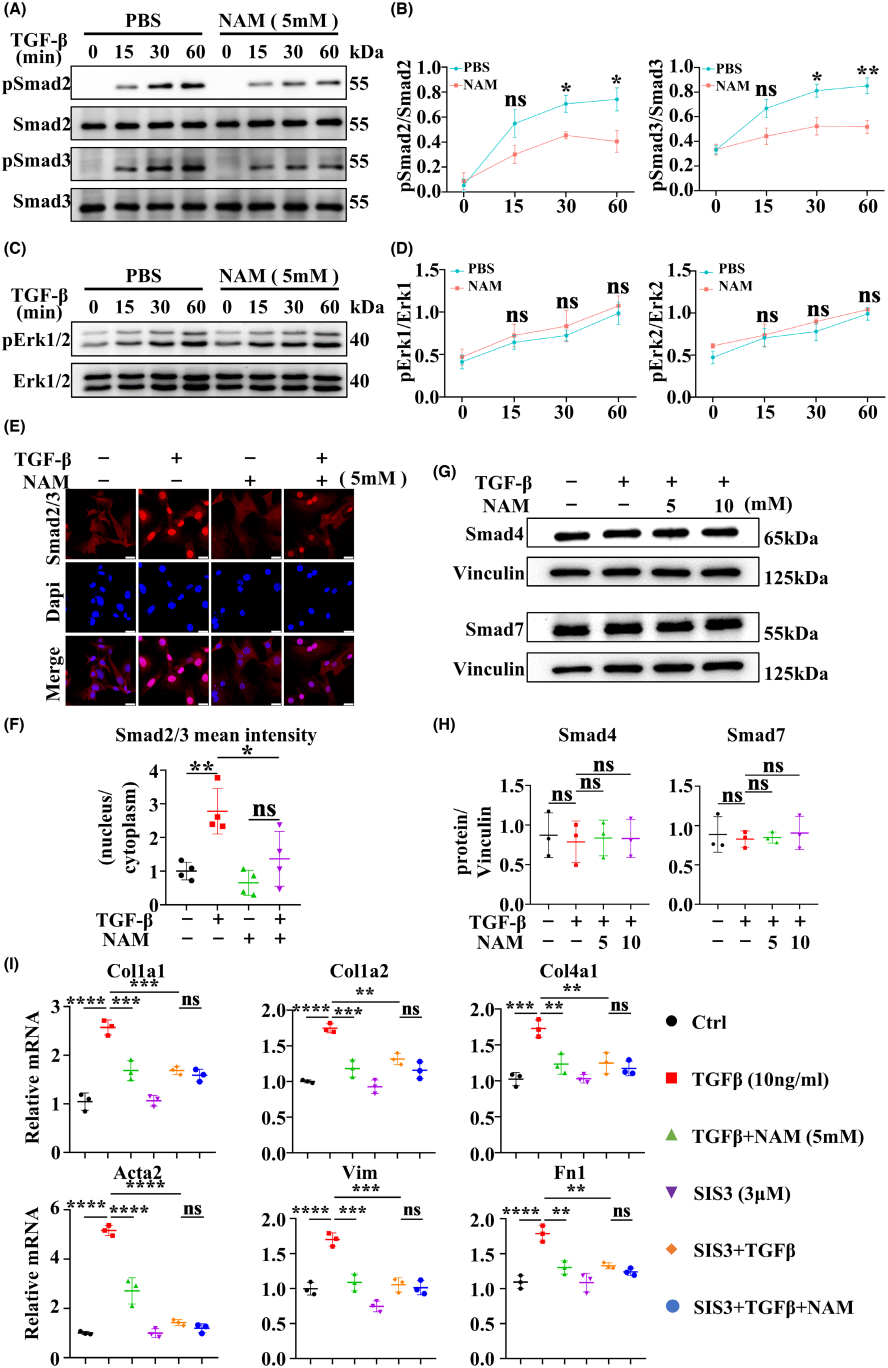
NAM mediated the inhibition of TGF‐β/SMADs pathway in vitro. MEFs were overnight preconditioned with NAM (5 mM) or SIS3 (3 μM), then cultured and incubated with TGFβ (10 ng/mL) for 0, 15, 30, 60 min, or 24 h. (A, B) WB results showing the expression of phospho‐SMAD2, phospho‐SMAD3, SMAD2, and SMAD2/3 proteins in vitro. (C, D) WB analysis of phosphorylated ERK1/2 and ERK1/2 protein expression in vitro. (E, F) Nuclear translocation of SMAD2/3 in primary MEFs was determined by IF (scale bar = 25 μm) and quantitative analysis was performed by ImageJ. (G, H) Expression of SMAD4 and SMAD7 proteins was determined by WB. (I) The expression of Col1α1, Col4α1, Fn1, Actα2, Col1α2, and Vimentin in vitro was detected by RT‐qPCR. ns indicates *p* > 0.05, *p < 0.05, **p < 0.01, ***p < 0.001, ****p < 0.0001. Significance of (B, D) was calculated using two‐way ANOVA followed by Sidak's multiple comparison test. Significance of (I) was calculated using two‐way ANOVA followed by Tukey's multiple comparison test. Significance of (F, H) was calculated using one‐way ANOVA followed by Tukey's multiple comparison test.

To gain further insights into the role of signaling pathways in NAM‐mediated suppression of fibrotic scar formation, we conducted experiments with MEFs. MEFs were treated with TGFβ for a series of times (0, 15, 30, and 60 min) following a 6‐h pre‐treatment with PBS or NAM. WB analysis revealed a significant time‐dependent reduction in the protein levels of phosphorylated SMAD2/3 following NAM treatment, demonstrating that NAM effectively suppressed the phosphorylation of SMAD2/3 in vitro (Figure 9A,B). Moreover, NAM exhibited the ability to inhibit the translocation of TGFβ‐stimulated SMAD2/3 into the nucleus, as shown in Figure 9E,F. However, NAM did not impact the ratio of TGFβ‐induced phosphorylated ERK1/2 (Figure 9C,D). To elucidate the mechanisms underlying NAM‐mediated inhibition of fibrotic scar formation, we assessed the protein expression of SMAD4 and SMAD7 via WB. Notably, NAM did not affect the protein expression of SMAD4 and SMAD7 even after 48 h of TGFβ stimulation (Figure 9G,H). Next, we used SIS3, an inhibitor of SMAD3 that inhibits its phosphorylation activation, to further confirm that the inhibitory effect of NAM on fibrotic scar formation is mediated by the TGFβ/SMADs pathway. RT‐qPCR results showed that SIS3 reduced the increased expression of fibrosis‐related genes induced by TGFβ, comparably to the effect of NAM alone. However, in the presence of SIS3, NAM did not show better therapeutic effects than NAM alone (Figure 9I). These collective findings strongly indicate that NAM restrains TGFβ/SMADs signaling by inhibiting the phosphorylation of SMAD2/3 and preventing their entry into the nucleus, thereby reducing fibrotic scar formation following SCI.

## 5 DISCUSSION

In previous studies, strategies to mitigate fibrotic scars in SCI predominantly revolved around molecular biology approaches.^53^, ^54^, ^55^, ^56^ The lack of a clear understanding of the cellular sources of scar‐forming fibroblasts and the absence of efficient molecular therapeutic targets have hindered advancements in SCI treatment and long‐term functional prognosis. The emergence of high‐throughput technologies encouraged researchers to delve into the molecular mechanisms underlying the pathogenesis of nervous system diseases,^57^, ^58^, ^59^ opening new avenues for SCI treatment. Transcriptomics predicts changes in gene expression and tells us “what can happen” or “what might happen”. Metabolomics, on the other hand, examines the functional changes in the expression of these genes and tells us “what is happening”, that is, what substances are changing as a result of life activities. Compared to transcriptomics, metabolomics is a more accurate reflection of the phenotype of an organism. Subtle changes in the transcriptome can be reflected and amplified by the metabolome. Metabolomics is located at the most downstream stage of systems biology, closest to the biological phenotype, and can establish a direct correlation with the biological phenotype. The combination of transcriptomics and metabolomics, that is, transcriptome–metabolome correlation analysis, can provide a deeper understanding of the mechanisms of cellular physiological activities at the molecular and biochemical levels. Therefore, the time point of 14 days after SCI was chosen for metabolomic analysis to better correlate with the biological phenotype at this stage.^60^ However, traditional metabolomics methods are limited to qualitative and quantitative analysis of specific metabolites. Single‐omics approaches often fall short of effectively elucidating the biochemical functions and related molecular mechanisms underlying disease occurrence, progression, and recovery.

In this research, we adopted a novel functional metabolomic strategy that combined transcriptomics and metabolomics analysis, aiming to identify functional metabolites that regulate fibrotic scar formation after SCI. We initially employed transcriptomics to characterize the significant upregulation of fibrosis‐related genes. Subsequently, precise targeted metabolomics allowed us to quantify local metabolites, and the results showed that nicotinate and nicotinamide metabolism were significantly enriched. Furthermore, we have observed some differences in niacinamide, fumaric acid, and other aspects between the last three mice in the SCI group and the other nine mice in SCI group (Figure 2B). Despite the fact that they underwent similar model construction, feeding conditions, and sample collection procedures, these mice exhibited distinct characteristics. We consider that these differences may be attributed to variations in the severity of secondary injury and pathological response. Given that metabolomics sits downstream in system biology, minor changes at the genetic and protein levels can be amplified through biological processes, resulting in significant differences in the metabolome.^60^ Besides, we cannot exclude the possibility of other unknown factors that may have influenced the experimental process. Subsequently, Spearman correlation analyses showed that NAM is a promising functional metabolite capable of regulating fibrotic scar formation. Subsequent function and mechanism experiments confirmed that NAM treatment inhibits fibrotic scar formation by downregulating the TGFβ/SMADs pathway. Our findings align with a study by Sally et al.,^61^ which reported that NAM inhibits invasive cell transformation during wound healing, offering potential therapeutic approaches for fibrotic eye diseases. This provides support for the feasibility of NAM in treating fibrotic scars in SCI.

In current studies, the use of metabolomics to uncover small‐molecule metabolites regulating various pathophysiological processes has attracted considerable attention.^62^, ^63^ Several studies have verified interactions between metabolites and inflammation. Luke O'Neill et al. found that pro‐inflammatory macrophages suppress their inflammatory response by producing endogenous small‐molecule metabolite itaconate.^64^ The anti‐inflammatory mechanism of itaconate involves the activation of Nrf2 through the alkylation of KEAP1, distinct from the action of exogenous small‐molecule anti‐inflammatory drugs. Moreover, their recent study,^65^ consistent with findings by Zecchini et al.,^66^ confirmed that the accumulation of fumaric acid in mitochondria can lead to inflammation associated with cancer and autoimmune diseases. Our previous research also revealed that five essential amino acids may regulate local inflammation in SCI through the brain–gut axis.^18^

In this study, we focused on the effects of local metabolites in injured spinal cord on the functional prognosis of mice after SCI. And our behavioral experiments such as BMS score and footprint analysis demonstrated that NAM could promote motor function recovery in injured mice (Figure 4). Notably, NAM has also shown promise in preventing neurological dysfunctions like Alzheimer's disease (AD) and Parkinson's disease (PD), as well as in treating depression and other psychological disorders.^67^, ^68^, ^69^, ^70^, ^71^ However, the results of the open field test suggest that SCI may be associated with anxiety‐like behavior in mice, and NAM treatment does not appear to mitigate this observed anxiety‐like behavior (Figure 4C). While the potential link between SCI and anxiety‐like behavior is intriguing, it is important to note that the underlying mechanisms may involve complex physiological and psychological changes, such as neurotransmitter imbalance, nerve pathway damage, and psychological stress.^72^, ^73^ In contrast, anxiety‐like behavior caused by Alzheimer's disease tends to focus more on changes in neurotransmitters and brain region function.^74^ Therefore, the improvement of anxiety‐like behavior does not solely depend on the balance of neurotransmitters but may also be influenced by various factors such as the animal's individual psychological state, environmental stimuli, and lifestyle following SCI. Although NAM has shown broad potential in the treatment of neurological dysfunction, we may need to further explore its therapeutic mechanisms and interactions with other factors when dealing with SCI‐induced anxiety‐like behaviors. Regardless, these findings underscore NAM's potential as a small‐molecule metabolite capable of crossing the blood–brain barrier (BBB) by promoting diffusion,^75^, ^76^ thus emerging as a promising candidate for treating spinal cord injuries.

In this study, we also focused on the relationship between local metabolite alterations and changes in the level of fibrosis‐related genes. Following SCI, metabolite levels at the injury site can more accurately reflect the local pathological condition compared to serum metabolites. Our results demonstrated that NAM can mediate the suppression of fibrosis‐related gene expression, leading to a reduction in the core lesion area and inhibition of fibrotic scar formation after SCI in vivo (Figure 5). Our results are consistent with Sally et al., who reported that NAM administration in an age‐related macular degeneration (AMD) model suppressed the expression of several types of genes, including TGFβ‐associated genes and collagen genes.^77^ Nie et al. demonstrated that NAM inhibited TGFβ1‐induced fibroblast proliferation and activation, resulting in downregulated expression of collagen I and fibronectin,^78^ which also coincides with our results. But it is worth noting that in our study results, vimentin did not show significant changes before and after NAM treatment in Figure 5C, whereas it exhibited significant changes in Figure 8A,C, as well as in Figure S5. We speculate that this may be due to the more complex in vivo environment. In vivo experiments involve a more intricate biological environment that comprises multiple cell types, intercellular interactions, signaling pathways, and potential unknown factors. In contrast, in vitro experiments are relatively simpler, involving only a single cell type or tissue, making it easier to observe significant differences in gene expression. This disparity could lead to less significant changes in vimentin gene expression in in vivo experiments, even when significant differences are observed in in vitro experiments. On the other hand, the significant change in vimentin protein levels in Figure 5D may be attributed to the protein's post‐translational modifications, such as phosphorylation and glycosylation, which alter its function and stability. Moreover, protein stability is influenced by numerous factors, including protease degradation. Furthermore, differences in experimental conditions and individual variations among mice may also affect gene expression and protein translation outcomes. What is more, our results indicate that NAM treatment suppressed the formation of fibrotic scars but did not affect the formation of glial scars. Glial scarring is a common and complex pathological process that occurs following SCI, which helps limit the spread of injury and protect neural tissue. Inhibiting glial scarring would be unfavorable for the regrowth of injured axons.^79^ Thus, NAM treatment preserves the beneficial effects of glial scarring while limiting the impediment of fibrotic scars on axonal regeneration. As of now, we have yet to explore the reasons why NAM treatment did not reduce glial scarring. However, it is undoubtedly a noteworthy finding, and we will conduct further investigations in subsequent studies to clarify NAM's impact on glial scarring post‐SCI.

Apart from that, our research results indicate that NAM has a protective effect on neuronal cell death after SCI (Figure 6), which is consistent with the research results of Zhu et al.^80^ Their research results confirm that nicotinamide mononucleotides (NMN) have a protective effect on traumatic brain injury. As a precursor of NMN, NAM may exert neuroprotective effects through the NAM—NMN—NAD^+^ axis. In addition, the IF results of NF200 also show that NAM treatment can promote axonal regeneration, but whether this is due to NAM's neuroprotective effect or its inhibitory effect on the formation of fibrotic scar is currently unclear, or both of them may work together to promote the extension of damaged axons across the damaged area.

**FIGURE 10:**
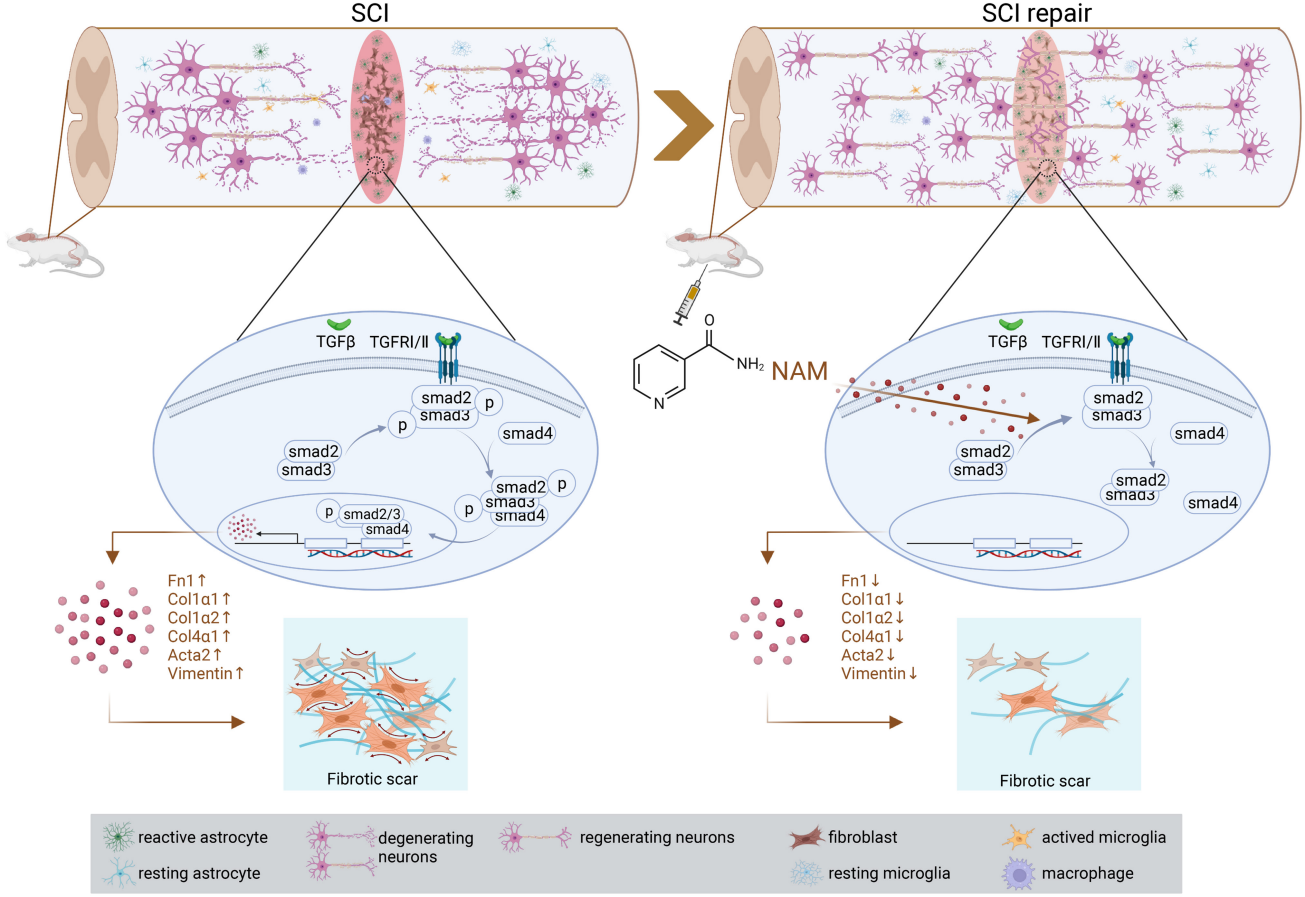
Mechanism diagram of NAM inhibiting fibrotic scar formation.

The therapeutic potential of NAM as a functional metabolite was further investigated by exploring the signaling pathways involved in fibrosis progression after SCI in vitro. Results of the functional experiments confirmed that NAM inhibited the formation of fibrotic scars after SCI by suppressing the TGFβ/SMADs signaling. Interestingly, our results were in line with findings by Sally et al., where it was found that NAM affected TGFβ signaling pathway in AMD models.^77^ In another study, it was observed that NAM attenuated TGFβ‐induced pro‐fibrotic changes in cultured proximal tubular cells.^81^ In addition, NAD^+^, an intermediate metabolite of NAM, inhibited the endothelial‐to‐mesenchymal transition induced by TGFβ in endothelial cells.^82^ The TGFβ/SMADs pathway has been shown to participate in multiple fibrotic disease models. Activation of this pathway by TGFβRI and TGFβRII stimulation enhances the recruitment and phosphorylation of SMAD2/3 to form the Smad2/3/4 complex. The complex then enters and accumulates in the nucleus where it binds to transcription factors and regulates expression of various genes.^83^ Our results showed that TGFβ‐induced phosphorylation of SMAD2/3 played a crucial role in the process of fibrosis, and NAM treatment reduced the ratio of phosphorylated SMAD2/3 and inhibited the nuclear translocation of the Smad2/3/4 complex. Notably, NAM did not affect the overall expression of SMAD4 and SMAD7 (Figures 7C and 9G), suggesting that NAM may act on the functional domains of TGFβRI/II or SMAD2/3 to regulate receptor‐ligand binding or recruitment of downstream protein complexes, and hence alter the ratio of phosphorylated SMAD2/3. The mechanism by which NAM drives the formation of anti‐fibrotic scar after SCI can be summarized as follows: NAM may affect the recruitment and/or phosphorylation of SMAD2/3 in cells by regulating the functional domains of TGFβR and/or SMAD2/3, thereby inhibiting the nuclear import of Smad2/3/4 complex. Through this process, it downregulates expression of fibrosis‐related genes and the expression of ECM‐related proteins, which inhibits fibrotic scar formation (Figure 10).

However, this study has some limitations. Although we found that NAM reduced the formation of fibrotic scar after SCI by inhibiting TGFβ/SMADs signaling pathway, the exact mechanism remains to be investigated. It is crucial to note that the reason for the reduced NAM content at the injured site is still unknown. Could this be due to local metabolic abnormalities? Alternatively, could it be associated with compromised nutrient absorption resulting from an imbalance in intestinal flora following SCI? Consequently, we will explore the underlying cause of the NAM content alteration in our future studies.

In conclusion, using a novel functional metabolomics strategy, we found that NAM is a potential functional metabolite that inhibits fibrotic scar formation in mice after SCI. Administration of NAM significantly reduced the fibrotic lesion area and promoted locomotor function recovery in mice with SCI, which suggested that NAM is a potential candidate for developing SCI therapeutic targets. Interestingly, consistent with previous reports, our results showed that NAM has therapeutic effects on various CNS diseases, indicating that NAM can be used to formulate new drugs for treating CNS diseases. Moreover, this innovative functional metabolomics strategy can be widely used to identify new effective therapeutic targets for various diseases.

## 6 CONCLUSION

A novel functional metabolomics strategy was developed to establish relationships between changes in gene expression and metabolic phenotypes, which identified NAM as a potential functional metabolite that suppressed fibrotic scar formation by suppressing the TGFβ/SMADs pathway in mice after SCI.

## AUTHOR CONTRIBUTIONS

Ce Zhang was the major contributor conducting experiments and toward the writing. Jianning Kang and Qiang Shao analyzed and interpreted the data. Wenjing Liu, Ying Zhang, Zhengxin Jin, and Nana Huang participated in figures production. Professor Ning participated in polishing and revising the article. All authors read and approved the final manuscript.

## FUNDING INFORMATION

Grant support was provided by the National Natural Science Foundation of China (Nos 81771346, 82071383, 82371392), Natural Science Foundation of Shandong Province, China (Key Project) (Nos ZR2020KH007), Natural Science Foundation of Shandong Province (Youth Program) (Nos ZR2020QH070, ZR2022QC222), the Taishan Scholar Youth Program of Shandong Province (tsqn201812156), and China Postdoctoral Science Foundation Funded Project (Project No. 2021M691225).

## CONFLICT OF INTEREST STATEMENT

None of the authors have a conflict of interest to disclose.

## CONSENT FOR PUBLICATION

All authors agree to the publication of this article.

## Supporting information


DataS1

FigureS1

FigureS2

FigureS3

FigureS4

FigureS5

TableS1

TableS2

TableS3

## Data Availability

The data that support the findings of this study are available from the corresponding author upon reasonable request.
