# Supplementary material for: Therapeutic application of nicotinamide: As a potential target for inhibiting fibrotic scar formation following spinal cord injury
Source: CNS Neurosci Ther. 2024 Jul 7;30(7):e14826. doi: 10.1111/cns.14826 (PMC11228357; doi:10.1111/cns.14826)

Full unedited blot for Figure 1

26625 marker

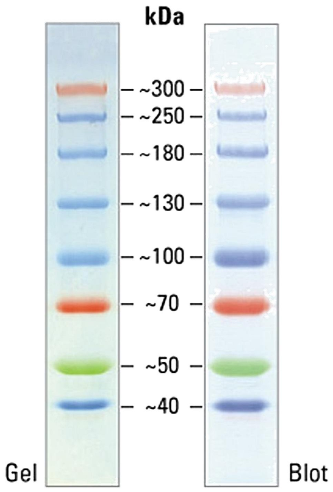

26616 marker

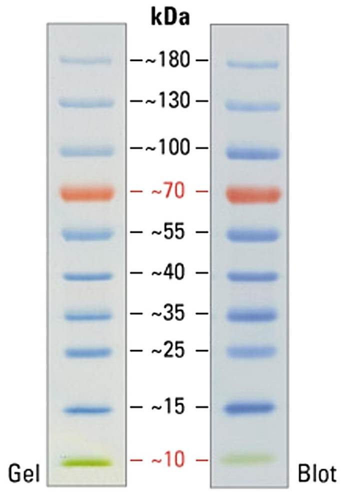

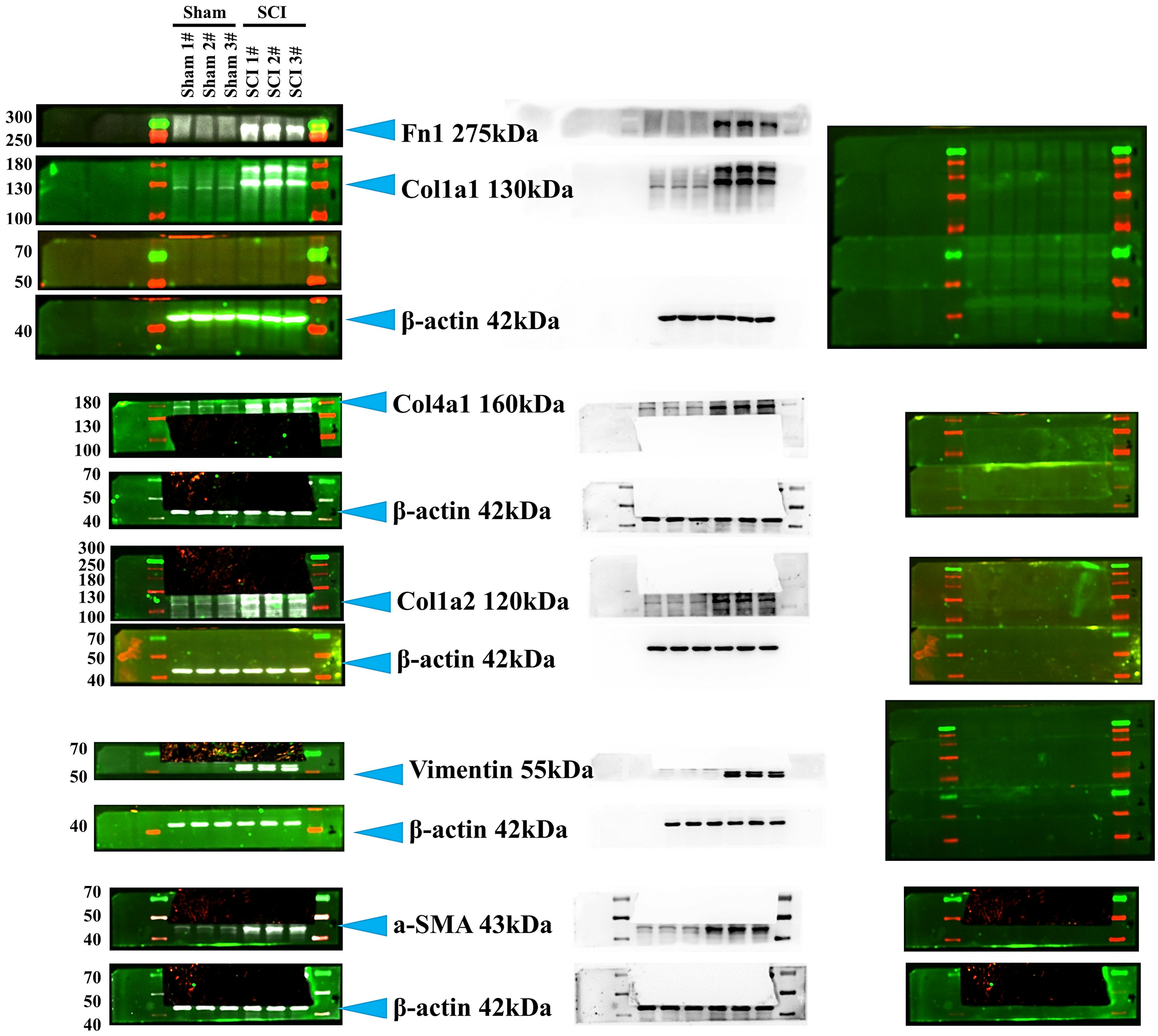

Full unedited blot for Figure 5

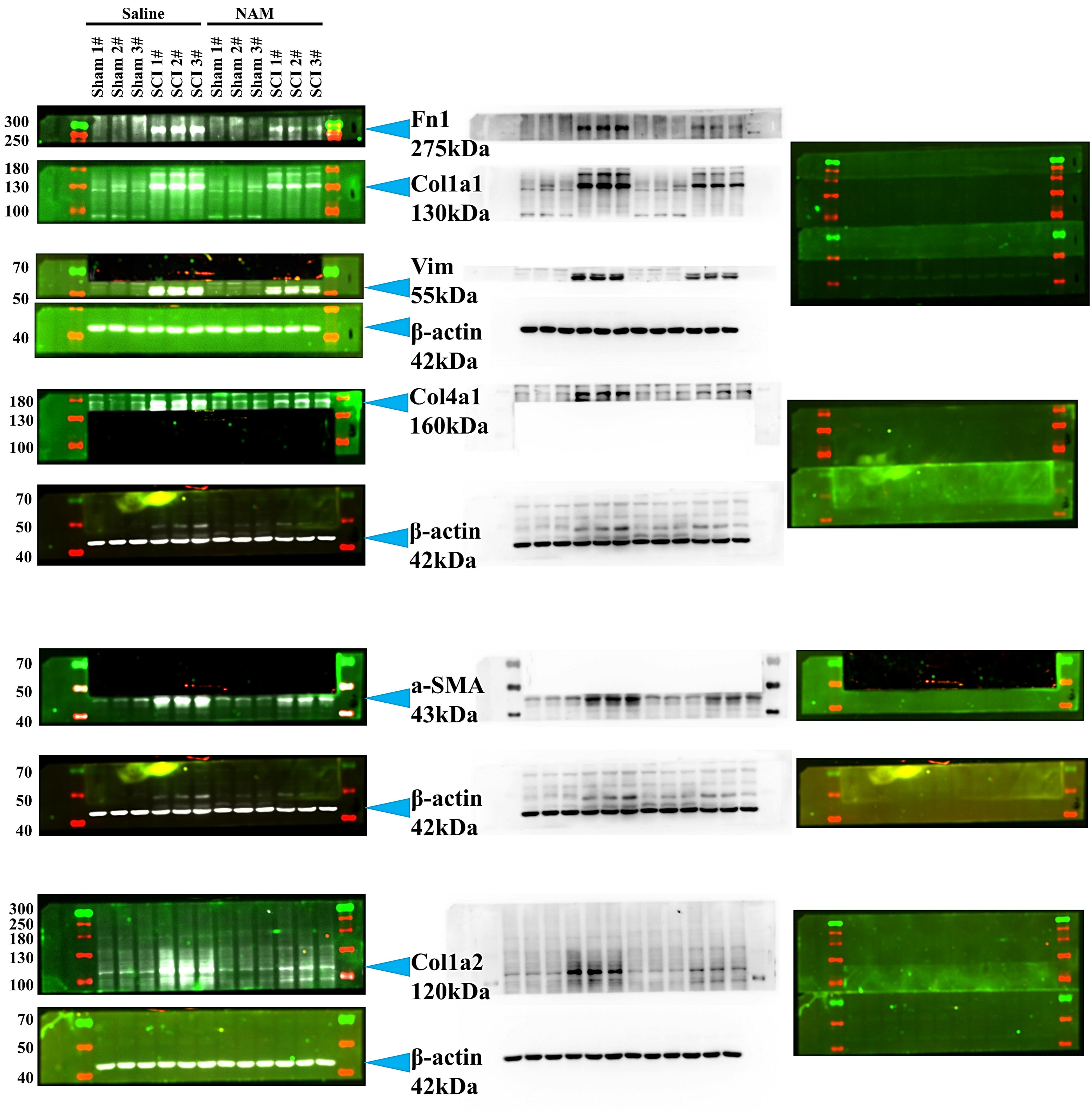

Full unedited blot for Figure 7

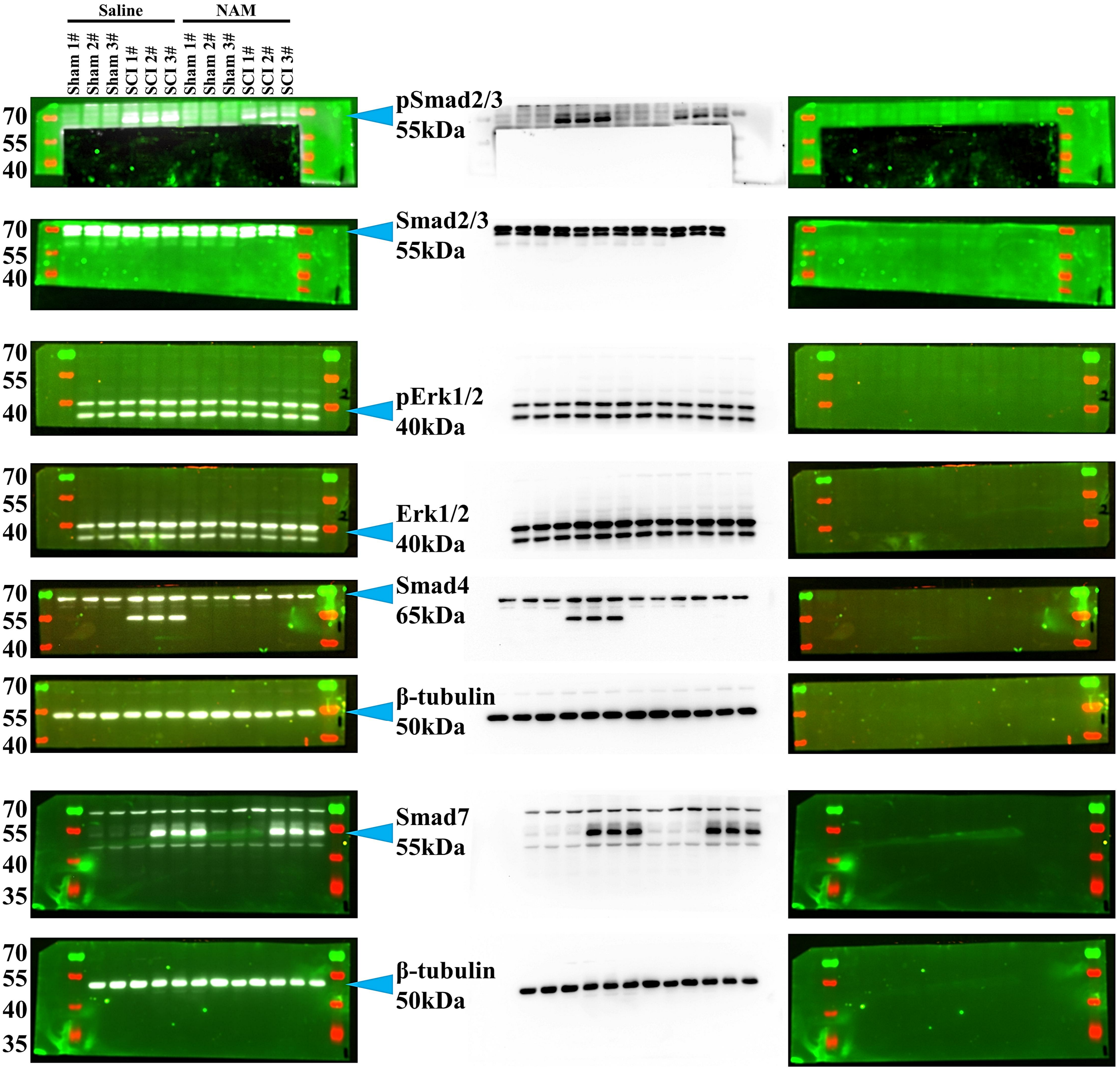

Full unedited blot for Figure 8

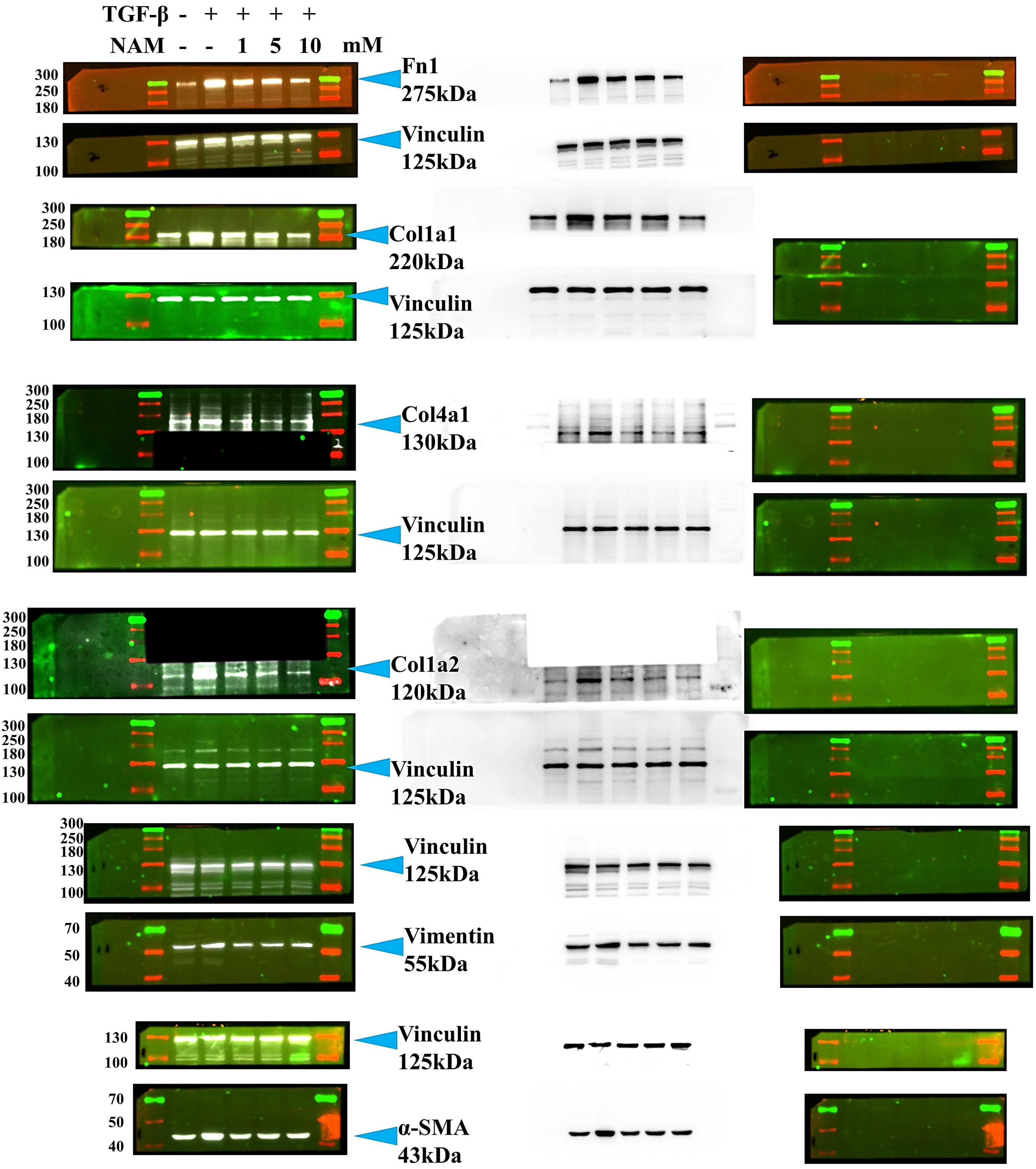

Full unedited blot for Figure 9

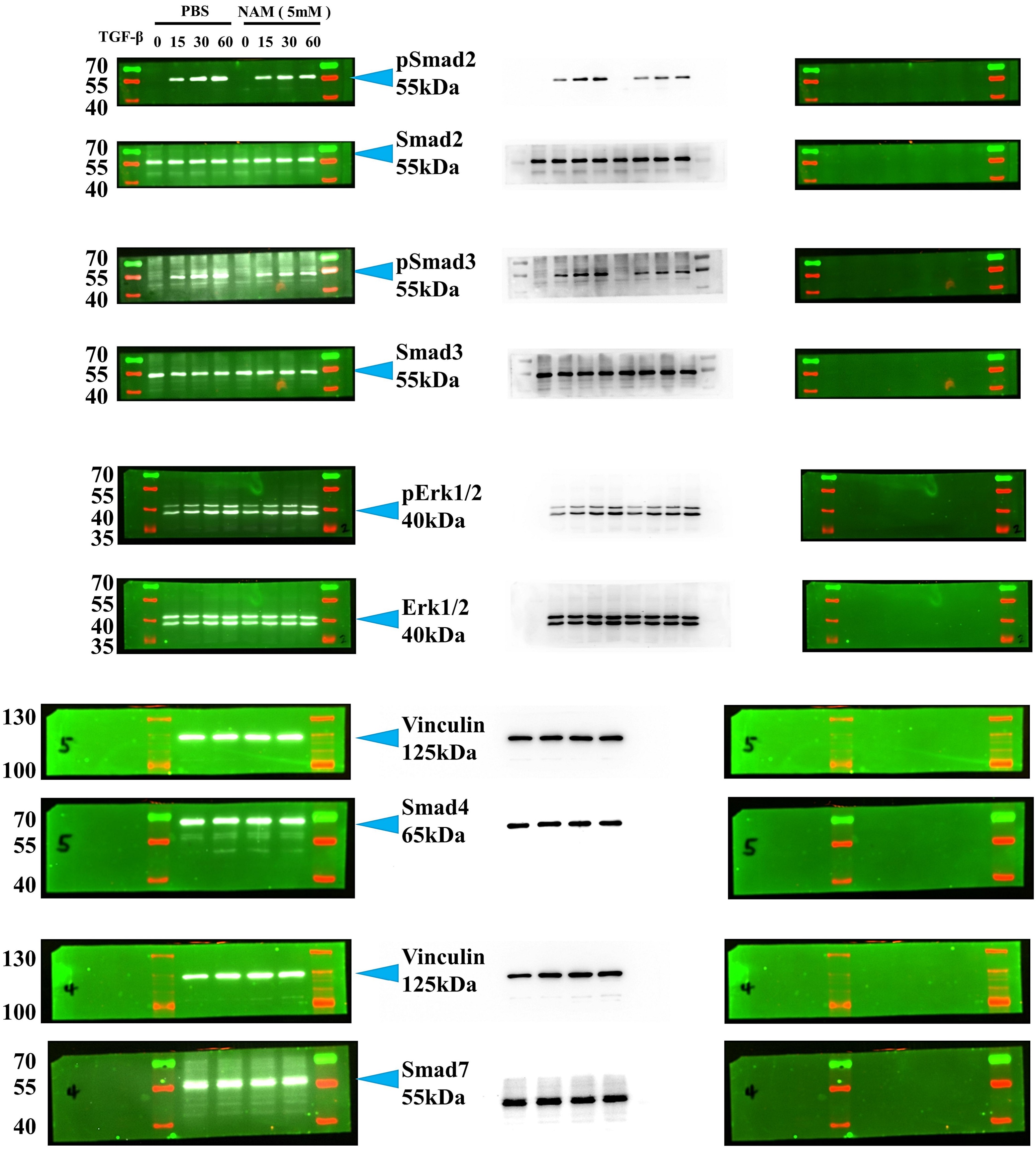

Supplement: Supplementary file 1 — DataS1 [file CNS-30-e14826-s005.pdf]
