# Supplementary figures and images for: Therapeutic application of nicotinamide: As a potential target for inhibiting fibrotic scar formation following spinal cord injury
Source: CNS Neurosci Ther. 2024 Jul 7;30(7):e14826. doi: 10.1111/cns.14826 (PMC11228357; doi:10.1111/cns.14826)

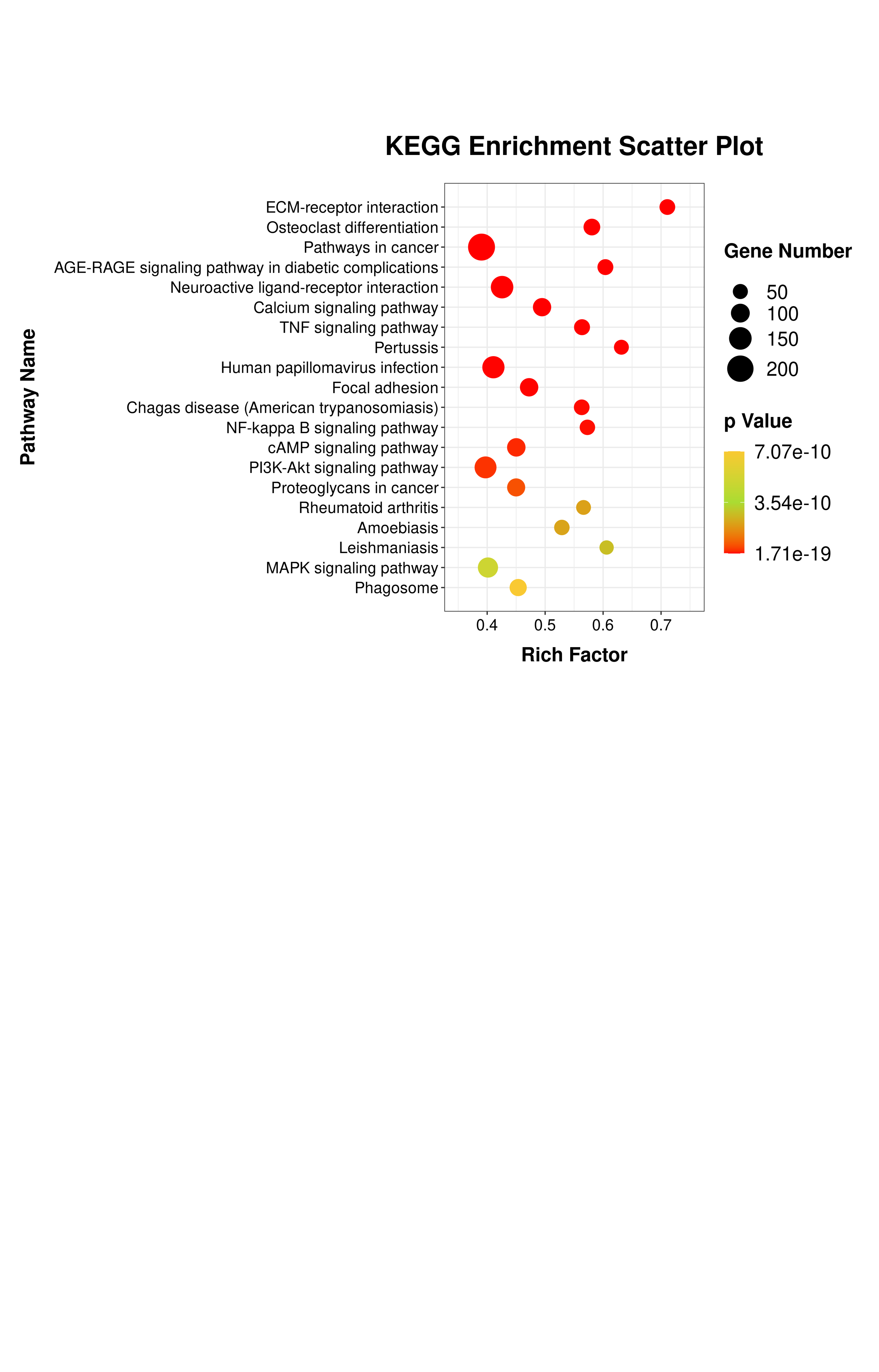

Supplement: Supplementary file 2 — FigureS1 [file CNS-30-e14826-s003.tif]

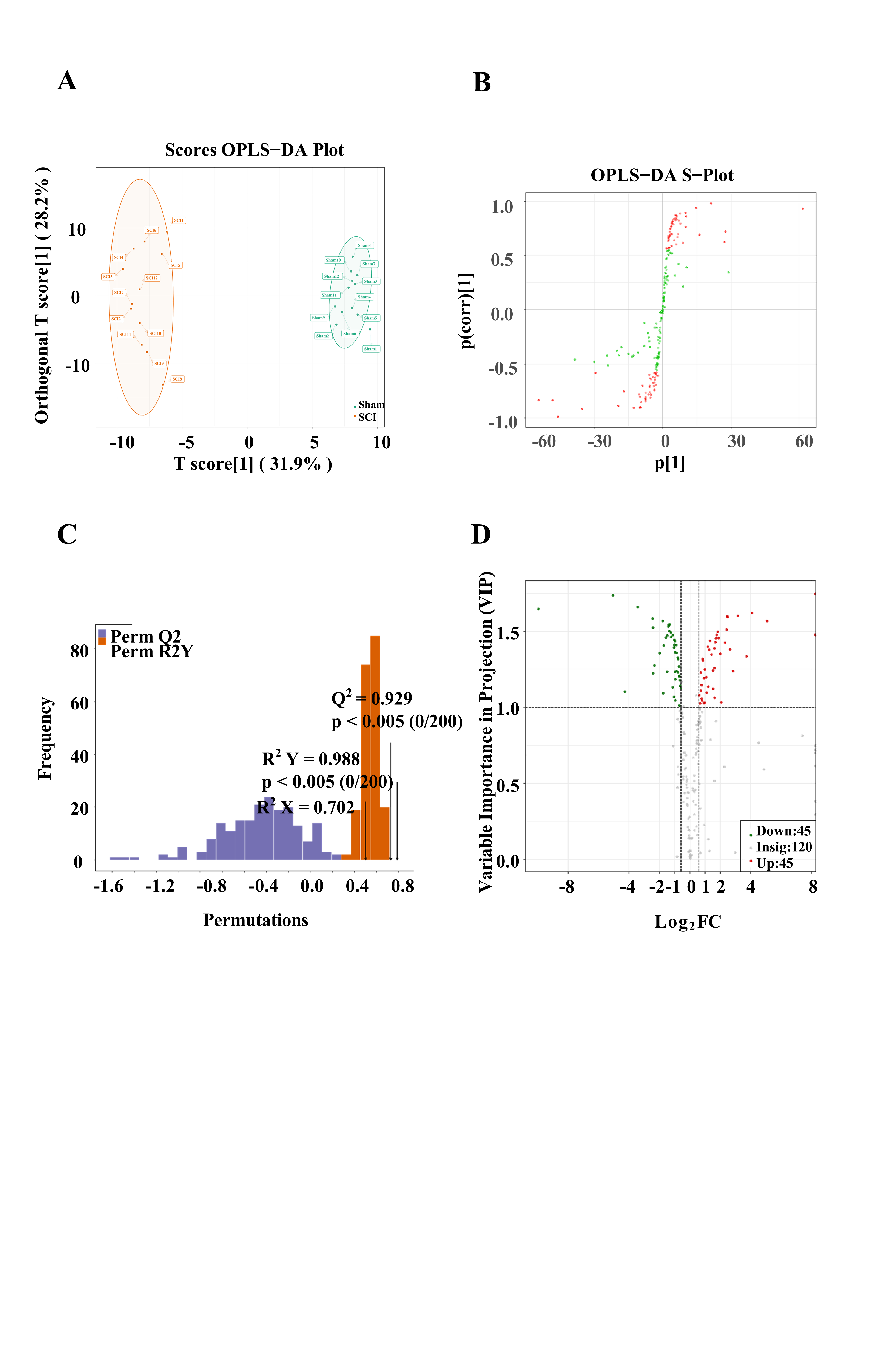

Supplement: Supplementary file 3 — FigureS2 [file CNS-30-e14826-s006.tif]

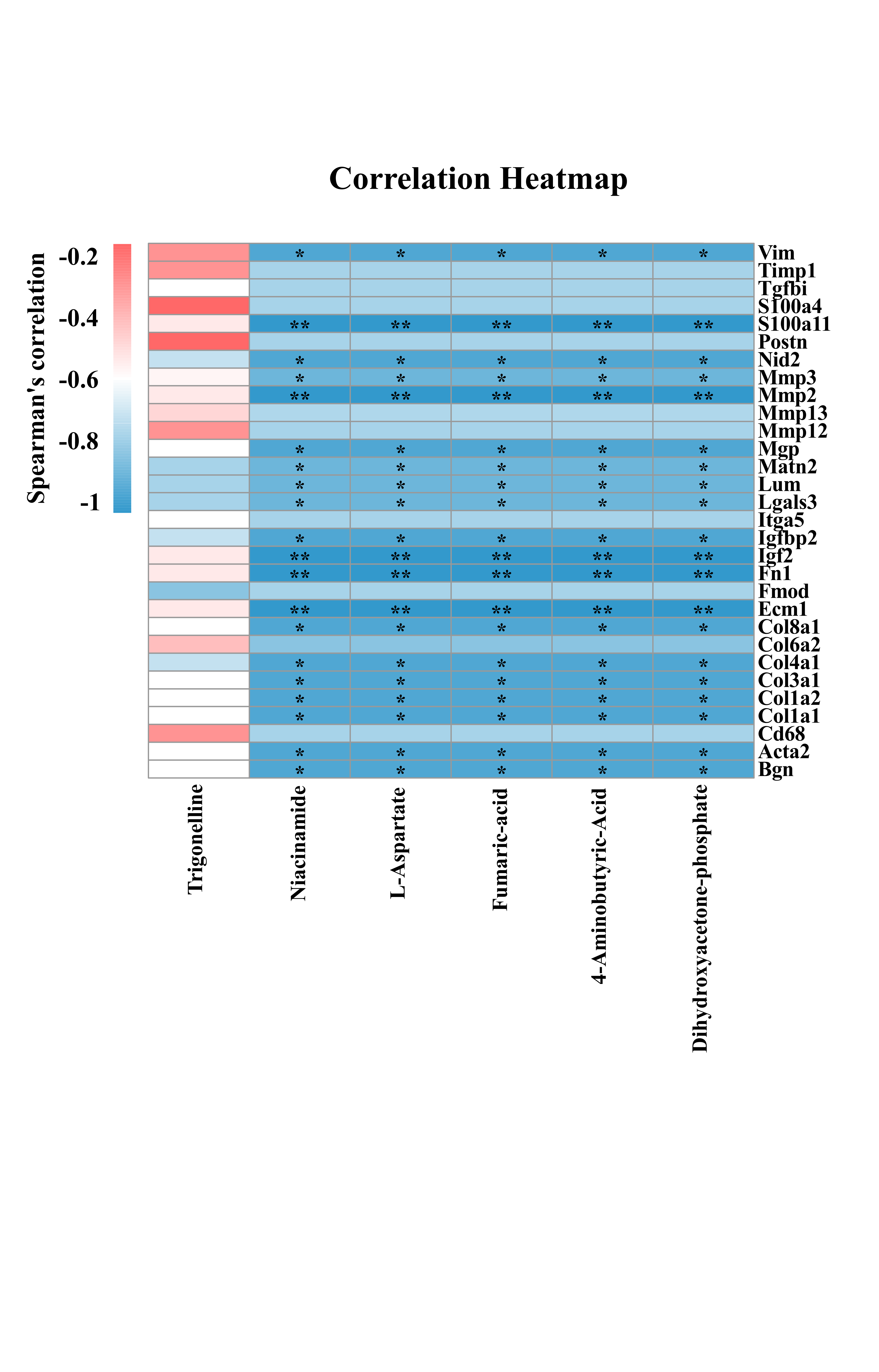

Supplement: Supplementary file 4 — FigureS3 [file CNS-30-e14826-s001.tif]

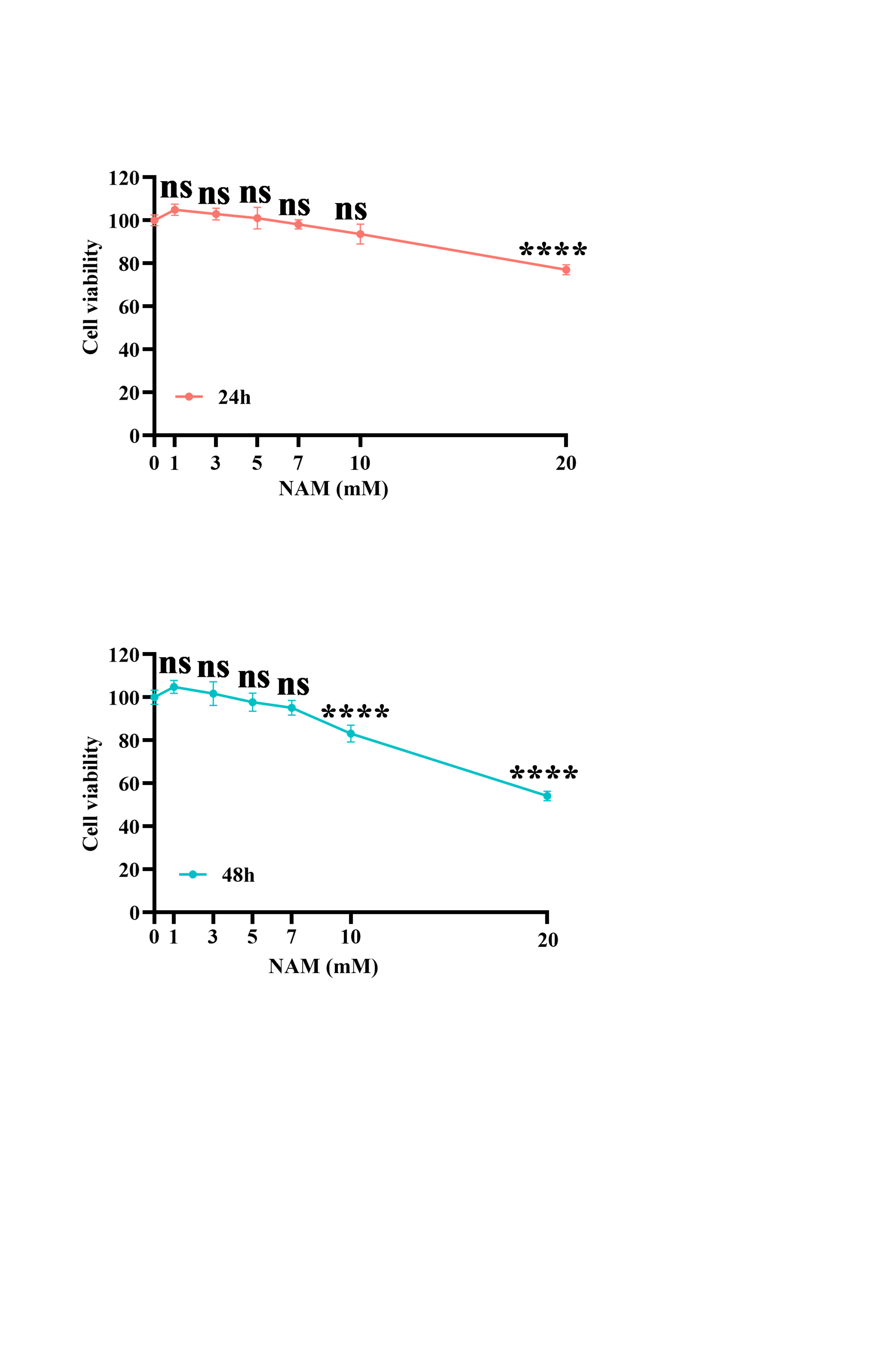

Supplement: Supplementary file 5 — FigureS4 [file CNS-30-e14826-s009.tif]

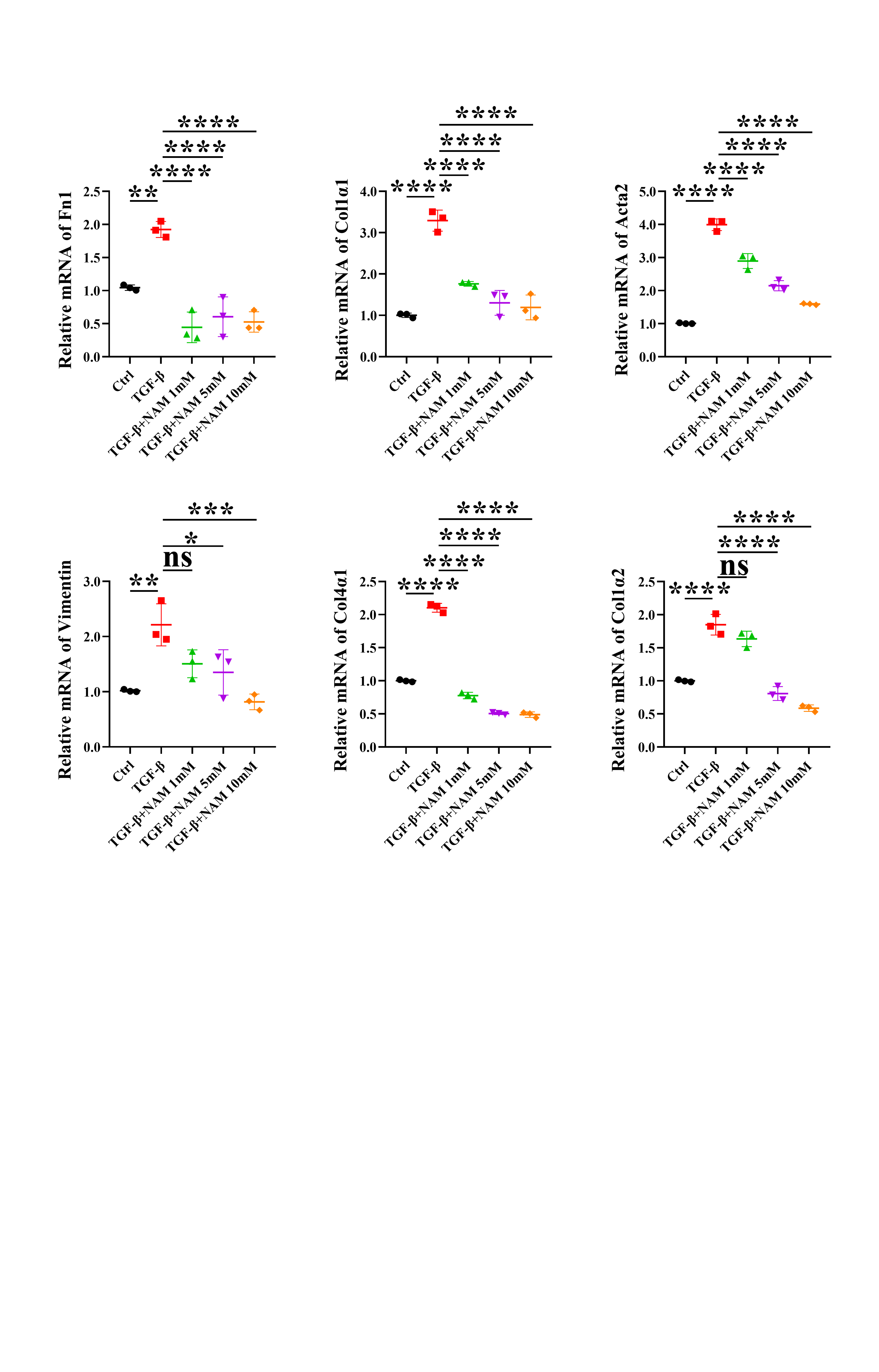

Supplement: Supplementary file 6 — FigureS5 [file CNS-30-e14826-s008.tif]
