# Supplementary material for: Therapeutic application of nicotinamide: As a potential target for inhibiting fibrotic scar formation following spinal cord injury
Source: CNS Neurosci Ther. 2024 Jul 7;30(7):e14826. doi: 10.1111/cns.14826 (PMC11228357; doi:10.1111/cns.14826)
Supplement: Supplementary file 7 — TableS1 [file CNS-30-e14826-s007.docx]

**Table S1. The primary and second antibodies used in this research.**

**Primary antibodies**

| **Name** | **Source** | **Application** | **Dilution** | **Manufacturers** |
| --- | --- | --- | --- | --- |
| Col1α1 | Rabbit | WB | 1:1000 | ABclonal Technology |
|  |  | IF | 1:100 |  |
|  | Rabbit | WB | 1:1000 | CST |
|  | Rabbit | WB | 1:2000 | Abcam |
| α-SMA | Rabbit | WB | 1:1000 | Servicebio |
|  |  | IF | 1:300 |  |
| p-SMAD2/3 | Rabbit | WB | 1:1000 | Cell Signaling Technology |
| SMAD2/3 | Rabbit | WB | 1:1000 |  |
|  |  | IF | 1:600 |  |
| p-ERK1/2 | Rabbit | WB | 1:1000 |  |
| ERK1/2 | Rabbit | WB | 1:1000 |  |
| Fn1 | Rabbit | WB | 1:1000 | Proteintech |
| Vimentin | Rabbit | WB | 1:5000 |  |
| Col1α2 | Rabbit | WB | 1:2000 |  |
| β-actin | Mus | WB | 1:10000 |  |
| β-tubulin | Rabbit | WB | 1:10000 |  |
| GFAP | Mus | IF | 1:200 |  |
| Vinculin | Rabbit | WB | 1:10000 | Abways Technology |
| SMAD4 | Mus | WB | 1:300 | Santa Cruz Biotechnology |
| SMAD7 | Mus | WB | 1:300 |  |
| Col4α1 | Rabbit | WB | 1:1000 | Immunoway |

**Second antibodies**

| **Name** | **Dilution** | **Manufacturers** |
| --- | --- | --- |
| Alexa Fluor 594-tagged goat anti-rabbit IgG | 1:200 | Abcam |
| Alexa Fluor 488-tagged goat anti-mouse IgG | 1:200 |  |
| Horseradish enzyme-labeled goat anti-rabbit IgG | 1:5000 | zsbio.com |
| Horseradish enzyme-labeled goat anti-mouse IgG | 1:5000 |  |
