# Supplementary material for: Therapeutic application of nicotinamide: As a potential target for inhibiting fibrotic scar formation following spinal cord injury
Source: CNS Neurosci Ther. 2024 Jul 7;30(7):e14826. doi: 10.1111/cns.14826 (PMC11228357; doi:10.1111/cns.14826)
Supplement: Supplementary file 8 — TableS2 [file CNS-30-e14826-s004.docx]

**Table S2. GO annotations for the RNA-sequence.**

**GO Enrichment**

| GO_ID | classification | GO_Term |
| --- | --- | --- |
| GO:0030054 | cellular_component | cell junction |
| GO:0035556 | biological_process | intracellular signal transduction |
| GO:0006954 | biological_process | inflammatory response |
| GO:0005886 | cellular_component | plasma membrane |
| GO:0098978 | cellular_component | glutamatergic synapse |
| GO:0005615 | cellular_component | extracellular space |
| GO:0005515 | molecular_function | protein binding |
| GO:0045202 | cellular_component | synapse |
| GO:0007155 | biological_process | cell adhesion |
| GO:0014069 | cellular_component | postsynaptic density |
| GO:0005576 | cellular_component | extracellular region |
| GO:0062023 | cellular_component | collagen-containing extracellular matrix |
| GO:0031012 | cellular_component | extracellular matrix |
| GO:0005604 | cellular_component | basement membrane |
| GO:0045211 | cellular_component | postsynaptic membrane |
| GO:0030198 | biological_process | extracellular matrix organization |
| GO:0005178 | molecular_function | integrin binding |
| GO:0045766 | biological_process | positive regulation of angiogenesis |
| GO:0005216 | molecular_function | ion channel activity |
| GO:0005518 | molecular_function | collagen binding |
| GO:0005581 | cellular_component | collagen trimer |
| GO:0006811 | biological_process | ion transport |
| GO:0016020 | cellular_component | membrane |
| GO:0005509 | molecular_function | calcium ion binding |
| GO:0035556 | biological_process | intracellular signal transduction |

GO: Gene Ontology
