# Supplementary material for: Therapeutic application of nicotinamide: As a potential target for inhibiting fibrotic scar formation following spinal cord injury
Source: CNS Neurosci Ther. 2024 Jul 7;30(7):e14826. doi: 10.1111/cns.14826 (PMC11228357; doi:10.1111/cns.14826)
Supplement: Supplementary file 9 — TableS3 [file CNS-30-e14826-s002.docx]

**Table S3. Primer for qRT-PCR**

| Primers for RT-qPCR | | |
| --- | --- | --- |
| Mus-Acta2 | F | 5'-TGAAGAGCATCCGACACT-3' |
|  | R | 5'-GCCTGAATAGCCACATACAT-3' |
| Mus-Fn1 | F | 5'-GACGTTGCAGAGCTATCCATTTC-3' |
|  | R | 5'-AGTGAATGAGTTGGCGGTGATAT-3' |
| Mus-Vimentin | F | 5'-GGATGTTGACAATGCTTCTCTGG-3' |
|  | R | 5'-TGGATCTCTTCATCGTGCAGTTT-3' |
| Mus-Col1a2 | F | 5'-GAAACATGGAAACCGAGGTGAAC-3' |
|  | R | 5'-AAGACCCTGCAATCCACTGTATC-3' |
| Mus-Col1a1 | F | 5'-GTGAGACAGGCGAACAAG-3' |
|  | R | 5'-CCAGGAGAACCAGGAGAA-3' |
| Mus-Col4a1 | F | 5'-AGGAACGACTACTCTTACTG-3' |
|  | R | 5'-CACTGCGGAATCTGAATG-3' |
| Mus-Gapdh | F | 5'-AAATGGTGAAGGTCGGTGTGAAC-3' |
|  | R | 5'-CAACAATCTCCACTTTGCCACTG-3' |

F: forward primer

R: reverse primer
